# Monocytes and interstitial macrophages contribute to hypoxic pulmonary hypertension

**DOI:** 10.1172/JCI176865

**Published:** 2025-01-30

**Authors:** Rahul Kumar, Kevin Nolan, Biruk Kassa, Neha Chanana, Tsering Palmo, Kavita Sharma, Kanika Singh, Claudia Mickael, Dara Fonseca Balladares, Julia Nilsson, Amit Prabhakar, Aastha Mishra, Michael H. Lee, Linda Sanders, Sushil Kumar, Ari B. Molofsky, Kurt R. Stenmark, Dean Sheppard, Rubin M. Tuder, Mohit D. Gupta, Tashi Thinlas, Qadar Pasha, Brian B. Graham

**Affiliations:** 1 Department of Medicine, University of California San Francisco, San Francisco, California, USA.; 2 Lung Biology Center, Zuckerberg San Francisco General Hospital, San Francisco, California, USA.; 3 Genomics and Molecular Medicine and; 4 Genomics and Genome Biology Unit, CSIR-Institute of Genomics and Integrative Biology, Delhi, India.; 5 Division of Pulmonary Sciences and Critical Care Medicine, Department of Medicine, University of Colorado Anschutz Medical Campus, Aurora, Colorado, USA.; 6 Cardiovascular Pulmonary Research Lab, University of Colorado School of Medicine, Aurora, Colorado, USA.; 7 Department of Laboratory Medicine, University of California San Francisco, San Francisco, California, USA.; 8 Department of Cardiology, GB Pant Institute of Post Graduate Medical Education and Research, New Delhi, India.; 9 Department of Medicine, Sonam Norboo Memorial Hospital, Leh, India.; 10 Institute of Hypoxia Research, Delhi, India.

**Keywords:** Inflammation, Vascular biology, Chemokines, Hypoxia, Monocytes

## Abstract

Hypoxia is a major cause of pulmonary hypertension (PH) worldwide, and it is likely that interstitial pulmonary macrophages contribute to this vascular pathology. We observed in hypoxia-exposed mice an increase in resident interstitial macrophages, which expanded through proliferation and expressed the monocyte recruitment ligand CCL2. We also observed an increase in CCR2^+^ macrophages through recruitment, which express the protein thrombospondin-1, which functionally activates TGF-β to cause vascular disease. Blockade of monocyte recruitment with either CCL2-neutralizing antibody treatment or CCR2 deficiency in the bone marrow compartment suppressed hypoxic PH. These data were supported by analysis of plasma samples from humans who traveled from low (225 m) to high (3500 m) elevation, revealing an increase in thrombospondin-1 and TGF-β expression following ascent, which was blocked by dexamethasone prophylaxis. In the hypoxic mouse model, dexamethasone prophylaxis recapitulated these findings by mechanistically suppressing CCL2 expression and CCR2^+^ monocyte recruitment. These data suggest a pathologic cross talk between 2 discrete interstitial macrophage populations, which can be therapeutically targeted.

## Abstract 2

CCL2-CCR2 signaling axis between resident and recruited lung interstitial macrophages contributes to hypoxic pulmonary hypertension.

## Graphical abstract

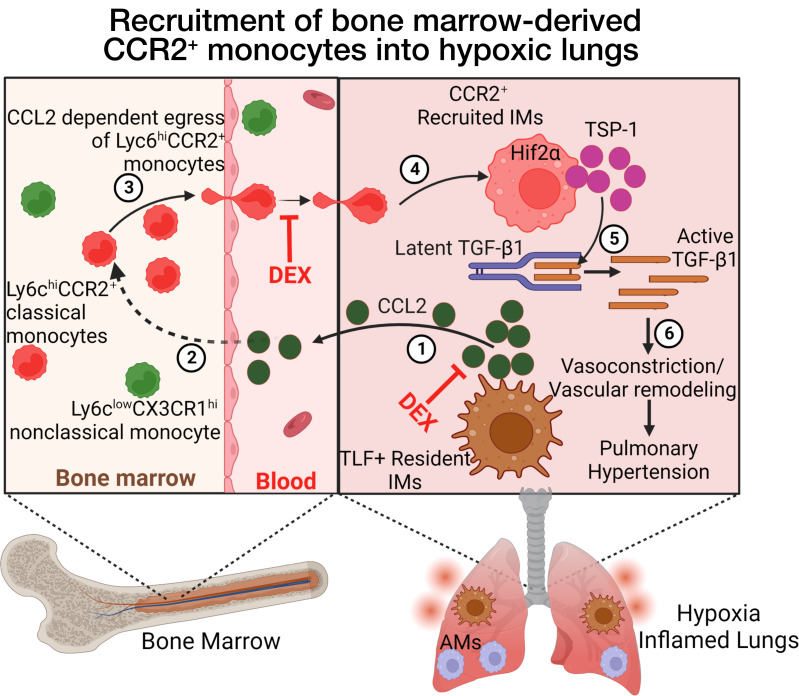

## Introduction

Hypoxia-induced pulmonary hypertension (hypoxic PH) is a common cardiovascular disease, caused by high altitude (HA) exposure and pulmonary diseases (1). Evidence indicates that inflammation significantly contributed to hypoxic PH pathogenesis in humans and experimental animal models. Bone marrow–derived (BM-derived) monocytes and macrophages (Mø) that infiltrate the pulmonary vasculature and the perivascular adventitial space promote pulmonary vascular remodeling in hypoxic PH (2–4). These monocytes and Mø likely contribute to disease pathogenesis by interacting with pulmonary vascular cells via proinflammatory cytokine and growth factor expression (5, 6). Interstitial Mø (IMs) are key cells that sense hypoxia and drive inflammatory lung pathologies. In hypoxic murine PH, BM-derived circulatory monocytes are recruited into the lungs where they become IMs (7–10). These recruited IMs express the matricellular protein thrombospondin-1 (TSP-1), which activates TGF-β, contributing to hypoxic PH by ρ-kinase–dependent mechanisms (7, 8).

Despite the potential role of IMs in PH pathobiology, mechanisms that underlie monocyte recruitment into the lungs in hypoxia to cause PH remain unclear, as the nature of the signaling is unknown, and the subpopulation of monocyte precursors that evolve to become TSP-1–expressing IMs remains undefined. Therefore, we aimed to elucidate inflammatory molecular signals that facilitate crosstalk between the BM compartment and the lungs, which we propose occurs early in hypoxia exposure. We also sought to characterize TSP-1^+^ IMs in the context of recently described self-renewing resident IMs, marked by cell surface proteins including folate receptor β (FOLR2) (11, 12).

Within the murine myeloid cell lineage, newly identified common monocyte progenitors (cMoPs), derived from monocyte and dendritic cell progenitors (MDPs) in the BM, generate classical monocytes. These classical monocytes are recognized by heightened expression of lymphocyte antigen 6c (Ly6c) and CC chemokine receptor-2 (CCR2). Subsequently, these Ly6c^hi^CCR2^+^ monocytes can transform into nonclassical monocytes (Ly6c^lo^CX3CR1^+^), characterized by reduced Ly6c levels and elevated fractalkine receptor (CX3CR1) expression (13, 14). Human monocyte subsets are similarly distinguished by CD14 and CD16 expression (15, 16). Monocyte subsets have been assigned specific roles based on studies of inflammatory conditions, with classical monocytes patrolling the vasculature and homing to inflamed sites, versus nonclassical monocytes promoting vascular homeostasis (17, 18). Monocyte precursor proliferation in the BM and subsequent monocyte migration to the blood and tissues is a tightly regulated process. CCR2 is the receptor required for classical monocyte migration into inflamed tissues. Among multiple chemokines, monocyte chemoattractant protein-1 (MCP-1, also known as CCL2) has the highest affinity to CCR2 (19, 20). Mounting evidence suggests CCL2’s pathologic role in inflammatory diseases (19, 21–26), including promoting monocyte extravasation from the blood and activation of recruited monocytes in inflamed tissues (27). However, data on cellular sources and roles of CCL2 in PH are limited and conflicting (28–30).

Understanding how disruptions to this delicate equilibrium in monocyte and Mø signaling may contribute to hypoxic PH represents a critical knowledge gap with important potential human implications. The clinical relevance of hypoxia and HA-induced inflammation is supported by the therapeutic use of dexamethasone (DEX), an antiinflammatory glucocorticoid. DEX is widely used to treat acute hypoxic lung diseases in HA travelers (31), but precise mechanisms underlying its clinical benefit are not clearly defined. Preclinical studies have shown that DEX improves and reverses experimental PH by reducing vascular smooth muscle cell proliferation (32, 33), but these studies used doses of DEX that were higher than what is used in clinical settings. While glucocorticoids suppress inflammatory cytokine transcription in human monocytes and Mø and downregulate chemokine expression such as CCL2 and CCL7 (34, 35), whether and how monocyte and Mø expression of these chemokines contribute to PH are unknown. Consequently, our study allows integrated investigation of human and mouse inflammatory responses to hypoxia with the aim of defining a plausible mechanism by which DEX prophylaxis offers protection against hypoxic PH by suppressing cytokine expression and the downstream inflammatory immune cascade, as hypothesized below.

We hypothesized that following hypoxia exposure, cMoPs in the BM proliferate and differentiate into classical monocytes. These classical monocytes then migrate to the lungs, where they become TSP-1–producing recruited lung IMs, driving TGF-β–mediated PH. Further, we sought to determine if prophylactic DEX modifies the CCL2 gradient required for classical monocyte recruitment into the lungs and attenuates hypoxic PH by suppressing TSP-1 and TGF-β1 in mice and humans exposed to HA.

## Results

### Hypoxia exposure results in experimental PH and increased cytokine expression in the lungs.

**Figure 1:**
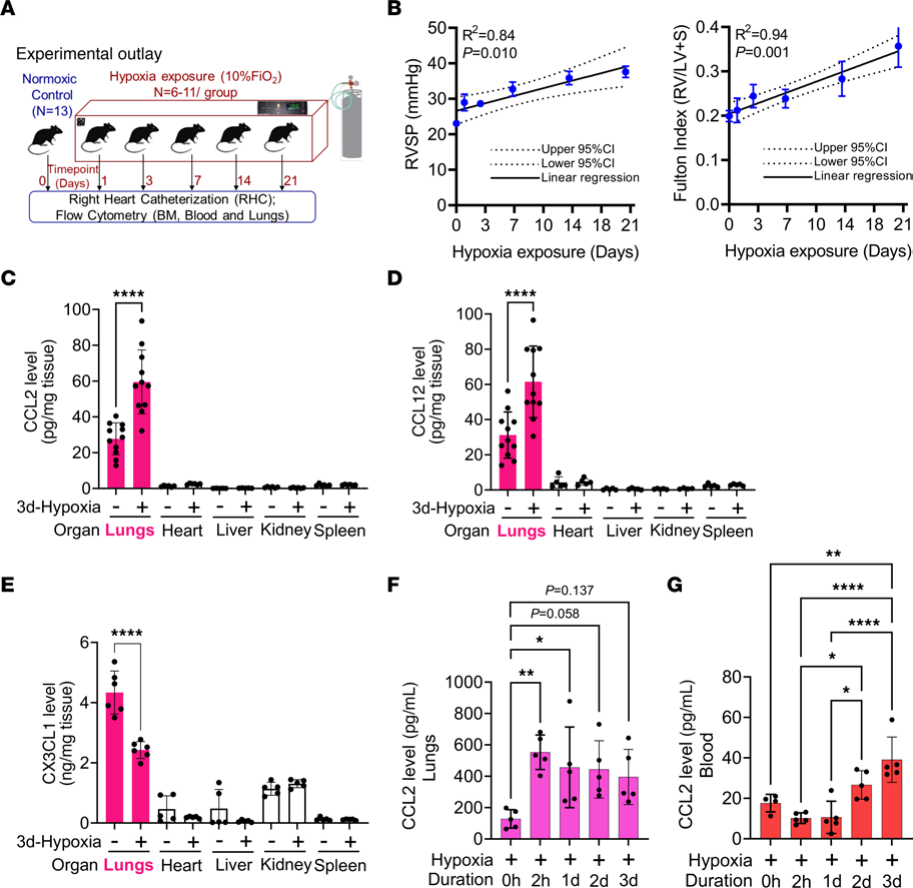
Exposure to high altitude results in PH and increased secretion of inflammatory classical monocyte ligands from the lungs. (**A**) Schematic showing hypoxia exposure time course in WT mice. Duration of hypoxia exposure is directly proportional to (**B**) RVSP and RV hypertrophy as measured by Fulton Index (*n* = 6–13/group). At 3 days of hypoxia, increased protein expression of classical monocyte ligands (**C**) CCL2 (*n* = 6–11/group) and (**D**) CCL12 (*n* = 6–11/group), whereas significantly lower levels of nonclassical monocyte ligand (**E**) CX3CL1 (*n* = 6/group) in the lungs. (**F**) Higher CCL2 gradient in lungs and in the (**G**) peripheral blood of WT mice following 3 days of hypoxia exposure (*n* = 5/group)**.** Data in all panels were obtained from female mice. Statistical analysis was conducted using ANOVA, followed by Tukey’s post hoc test. *P < 0.05, **P < 0.01, ****P < 0.0001. *n* = number of animals, mean ± SD; CI, confidence interval.

We performed serial hemodynamic measurements on mice exposed to hypoxia at various time points (Figure 1A), and observed progressive elevation in right ventricular systolic pressure (RVSP) and right ventricular hypertrophy (RVH), as determined by the Fulton Index (Figure 1B and Supplemental Figure 1, A–C; supplemental material available online with this article; https://doi.org/10.1172/JCI176865DS1).

Multiorgan assessment of inflammatory cytokines and ligands in unexposed and 3-day hypoxia-exposed mice revealed a significant increase in 2 CCR2 ligands, CCL2 (MCP-1) and CCL12 (MCP-5) (Figure 1, C and D) in the hypoxia-exposed mouse lungs, as compared with lungs from unexposed mice and other organs. In an organ-specific independent comparison, heart and liver of WT mice exposed to hypoxia displayed elevated levels of CCL2 (Supplemental Figure 1D). There was no difference in another CCR2 ligand, CCL7 (MCP-3; Supplemental Figure 1E). We also observed a decrease in the CX3CR1 ligand CX3CL1 (fractalkine) in the lungs of hypoxia-exposed mice at this early timepoint (Figure 1E). In addition, the blood of hypoxia-exposed mice had significantly elevated CCL2, CCL12, and CCL7 (Supplemental Figure1, F–H) compared with normoxic mice. Time course–based analysis showed a higher concentration of the ligand CCL2 in the lungs as early as 2 hours after hypoxia exposure, whereas blood levels of CCL2 increased starting 2 days after hypoxia exposure (Figure 1, F and G).

### Classical monocytes serve as the precursor of recruited inflammatory IMs.

**Figure 2:**
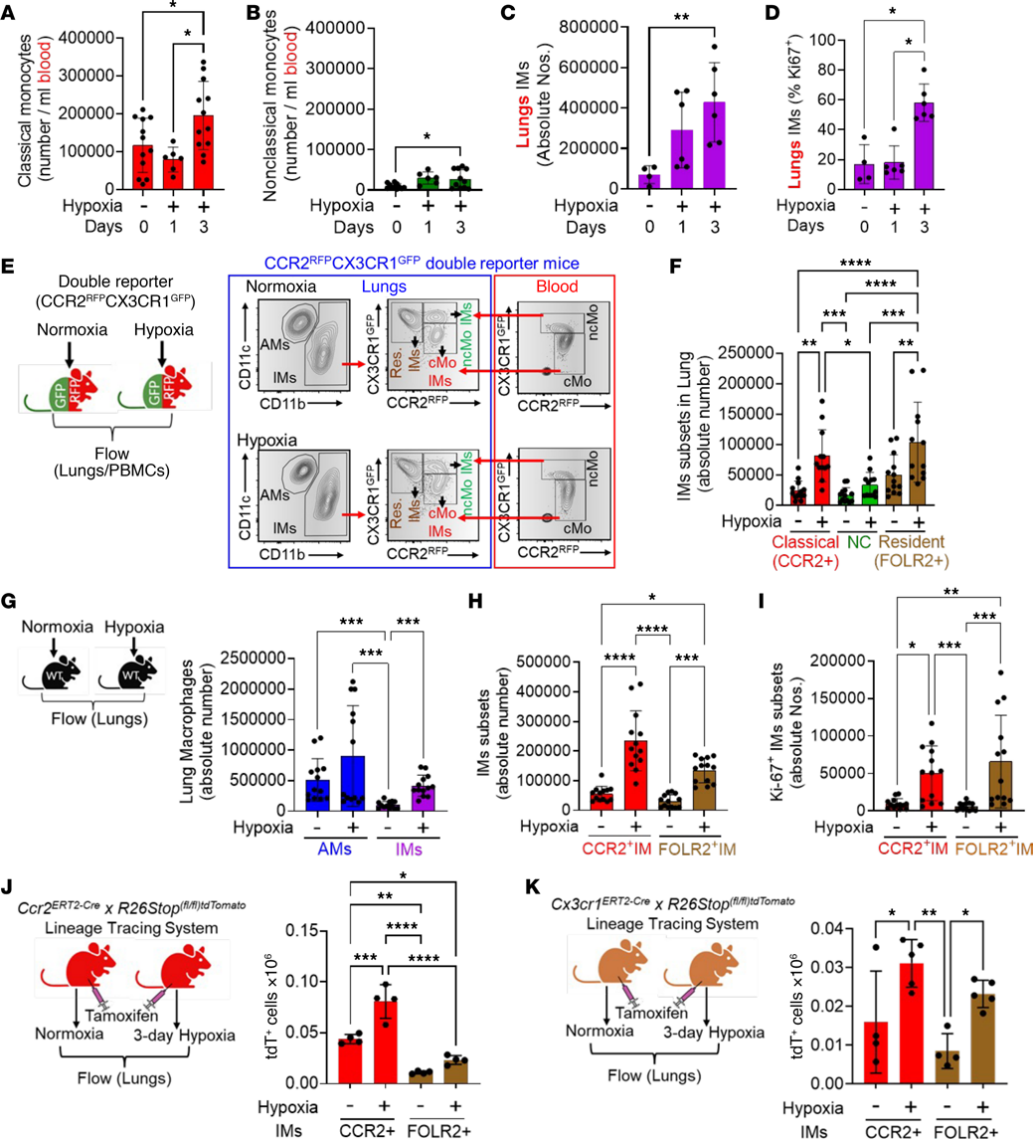
Classical monocytes serve as a precursor of inflammatory IMs following hypoxia exposure. Time course–based flow cytometry analysis showed increased numbers of (**A**) classical and (**B**) nonclassical monocytes in circulation and (**C**) recruitment of IMs with (**D**) higher proliferation after 3 days of hypoxia in lungs (*n* = 6/group; all female mice). (**E**) Double reporter (*Ccr2^RFP^Cx3cr1^GFP^*) mice flow cytometry revealed extravascular IMs subsets: RFP^+^ (recent recruitment from the circulatory CCR2^+^ monocytes) and another RFP^–^ GFP^+^ resident (FOLR2^+^) IMs (representative image of *n* = 12–13/group). (**F**) Quantitative analysis showed significant increases in CCR2^+^ IMs and FOLR2^+^ resident IMs following hypoxia exposure (*n* = 13 in Nx; 8 female, 5 male; *n* = 12 in Hx, 8 female, 4 male). (**G**) WT mice flow cytometry showed elevated total IMs after hypoxia (*n* = 7/group; all female mice). (**H**) Both CCR2^+^ IMs and FOLR2^+^ resident IMs increased significantly following hypoxia exposure (*n* = 7/group; all female mice). (**I**) FOLR2^+^ IMs were highly positive for the proliferation marker Ki-67 after hypoxia exposure (*n* = 7/group; all female mice). (**J**) *Ccr2^ERT2–Cre^ x R26Stop^(fl/fl)tdTomato^* lineage tracing system efficiently labeled CCR2^+^ IMs with tdT in hypoxia compared with FOLR2^+^ IMs following tamoxifen injection (*n* = 4/group; 2 male, 2 female in Nx; 2 male, 2 female in Hx group). (**K**) *Cx3cr1^ERT2–Cre^ x R26Stop^(fl/fl)tdTomato^* lineage tracing system efficiently marks resident FOLR2^+^ IMs with tdTomato (tdT); circulatory CCR2^+^ monocytes also showed slightly higher tdT^+^ labeled in hypoxia compared with normoxia (*n* = 4–5/group; 2 male, 2 female in Nx; 2 male and 3 female in Hx group). ANOVA followed by Tukey’s post hoc test was conducted for Panels **A**, **B**, **F**, **J**, and **K**. Kruskal-Wallis ANOVA followed by Dunn’s post hoc test was used for panels **C**, **D**, and **G**–**I**. mean ± SD plotted. ^#^*P* < 0.05, **P < 0.01, ***P < 0.001, ****P < 0.0001. *n*, number of animals; NC, nonclassical; Nos., number; IMs, interstitial macrophages.

We found a significant increase in both classical and nonclassical monocytes in the intravascular compartment 3 days after hypoxia exposure compared with unexposed WT mice (Figure 2, A and B). We also observed a significantly higher number of extravascular lung IMs with increased proliferation (Ki67^+^) (Figure 2, C and D). We did not find any difference in the number or proliferation of alveolar macrophages (AMs; Supplemental Figure 2, A and B).

To gain insight into the potential precursor of these recruited IMs, we challenged *Ccr2^+/RFP^Cx3cr1^+/GFP^* mice with hypoxia, in which classical CCR2^+^ monocytes are marked by RFP, and nonclassical CX3CR1^+^ monocytes are marked by GFP. We found that the extravascular IMs were largely RFP^+^, consistent with a classical or CCR2^+^ monocyte precursor (Figure 2, E and F). There was another IM population largely negative for CCR2 but positive for CD11b and CX3CR1 in the lungs. These CCR2^–^ IMs were FOLR2^+^ and, therefore, likely represent resident, nonrecruited IMs (Figure 2E), based on recent reports (11, 12), which characterized resident IMs as expressing FOLR2, and recruited IMs expressing CCR2. Both recruited CCR2^+^ and resident FOLR2^+^ IMs significantly increased in number with hypoxia (Figure 2F). We also looked for FOLR2 versus CCR2 expression on IMs in WT mice and observed that both FOLR2^+^ resident IMs and CCR2^+^ recruited IMs significantly increased in number following hypoxia exposure (Figure 2, G and H). We observed higher proliferation (Ki67^+^) of FOLR2^+^ resident IMs specifically, compared with CCR2^+^ recruited IMs following hypoxia exposure (Figure 2I). Due to the influx of CCR2^+^ IMs (increasing from making up 9% of all IMs to 41%), there was a relative reduction in the fraction of FOLR2^+^ IMs (decreasing from 88% to 54%, Supplemental Figure 2C).

We observed increased lung CCL2 as early as 2 hours after hypoxia (Figure 1G). However, no change in interstitial macrophages was observed at this early time point (Supplemental Figure 2D), suggesting that lung CCL2 expression precedes CCR2^+^ IM recruitment. The gating strategy to characterize monocytes and monocyte-derived IMs in the blood and lungs of WT and *Ccr2^+/RFP^Cx3cr1^+/GFP^* mice is shown in Supplemental Figures 3–6.

To further detail the origin of CCR2^+^ IMs in hypoxic PH, we utilized lineage-tracing models for resident IMs by employing both *Ccr2*^ERT2–Cre^ × *R26Stop^(fl/fl)tdTomato^* and *Cx3cr1-*^ERT2–Cre^ × *R26Stop^(fl/fl)tdTomato^* mice. Prior studies reported that administering tamoxifen to *Cx3cr1-*^ERT2–Cre^ × *R26Stop^(fl/fl)tdTomato^* mice results in labeling nonrecruited, resident IMs expressing CX3CR1 with tdTomato (tdT) fluorescence, without marking circulating Ly6c^hi^CCR2^+^ monocytes, whereas tamoxifen administration to *Ccr2*^ERT2–Cre^ × *R26Stop^(fl/fl)tdTomato^* mice selectively labels circulating Ly6C^+^CCR2^+^-derived recruited IMs with tdT, without labeling noncirculatory-derived resident FOLR2^+^ IMs (36–38). As illustrated in the Figure 2J schematic, 1 week following tamoxifen administration, experimental mice were exposed to 3 days of hypoxia, while control mice remained in normoxia, and flow cytometry was used to distinguish tdT-labeled from unlabeled cells. Using *Ccr2^ERT2–Cre^* × *R26Stop^(fl/fl)tdTomato^* mice, we observed more tdT^+^ cells in the CCR2^+^ IMs after hypoxia and little change in the number of tdT^+^ resident FOLR2^+^ IMs (Figure 2K). The increase in FOLR2^+^ IMs was largely from tdT^–^ populations, whereas there was little change in tdT^–^ CCR2^+^ IMs with hypoxia exposure (Supplemental Figure 7A).

In contrast, using the *CX3CR1^ERT2–Cre^* × *R26Stop^(fl/fl)tdTomato^* mice revealed a higher number of tdT^+^ cells associated with resident FOLR2^+^ IMs after 3 days of hypoxia (Figure 2K), consistent with proliferation of resident CX3CR1^+^ IMs in situ, without a requirement for recruitment. We also observed more tdT^+^ recruited CCR2^+^ IMs, which could be due, in part, to contributions from circulating CX3CR1-expressing nonclassical monocytes, or, more likely, cells that express both receptors, as we observed circulating cells expressing both CX3CR1 and CCR2 (Figure 2E). There was a significant increase in tdT^–^ cells within the CCR2^+^ IMs, consistent with recruitment of classical monocytes (Supplemental Figure 7B). The gating strategies are in Supplemental Figures 8 and 9. In sum, these findings suggest a prominent intravascular origin for CCR2^+^ IMs, largely from CCR2-expressing classical monocytes.

### Imaging reveals increased perivascular macrophages in hypoxic lungs.

Qualitative and quantitative imaging of *Ccr2^+/RFP^Cx3cr1^+/GFP^* reporter mice revealed significant alterations in IM subsets and vascular remodeling in the lungs following hypoxia exposure. Both CCR2^+^ and CX3CR1^+^ cells were found in close proximity to vessels (Supplemental Figure 10, A–D). Under hypoxic conditions, we observed a marked increase in the percentages of CCR2^+^ and CX3CR1^+^ IMs within 10 μm and 10–20 μm of vascular regions, respectively, compared with normoxic controls, with a particularly high density of both populations under 10 μm from vessels (Supplemental Figure 10, E–H). We observed no change in CCR2^+^ and CX3CR1^+^ cells 20–40 μm from vessels in hypoxia-exposed lungs, suggesting the increase in IM density is particularly adjacent to vessels (Supplemental Figure 10, I and J). We also observed significantly increased α-smooth muscle actin (α-SMA) volume, consistent with vascular remodeling (Supplemental Figure 10K). Of note, compared with flow cytometry data using *Ccr2^+/RFP^Cx3cr1^+/GFP^* reporter mice and analysis of lung single cell RNA sequencing (scRNASeq) data from recently published work (39) (Figure 2E and Supplemental Figure 10L), we align CX3CR1-expressing single-positive cells largely with FOLR2^+^ IMs, whereas CCR2-expressing cells align largely with CCR2^+^ IMs. These findings highlight the impact of hypoxia on perivascular IM density and spatial localization.

### Hypoxia increases monocyte production by the bone marrow compartment.

**Figure 3:**
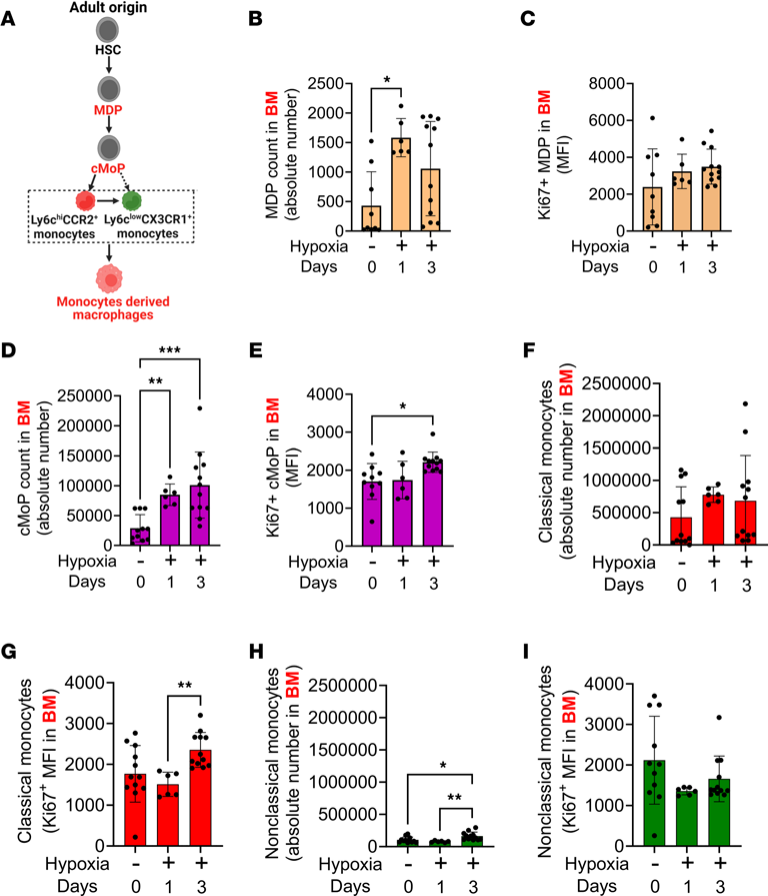
Higher number and increased proliferation of classical monocytes and their precursors in BM following hypoxia exposure. (**A**) Schematic showing myeloid progenitor phylogeny in the BM compartment. Hypoxia exposure leads to (**B**) a higher number and (**C**) a trend toward higher proliferation of MDPs (*n* = 6–12/group). Hypoxia results in (**D**) a higher number (*n* = 6–12/group) and (**E**) increased proliferation of cMoP in the BM compartment (*n* = 6–12/group). There was (**F**) no difference in number (*n* = 6–12/group) but (**G**) higher proliferation of classical monocytes (*n* = 6–12/group) following 3 days of hypoxia exposure. There was a (**H**) higher number (*n* = 6–12/group), with no change in the (**I**) proliferation of nonclassical monocytes in hypoxia (*n* = 6–12/group). For all panels, data were not normally distributed. Therefore, the Kruskal-Wallis ANOVA test, followed by Dunn’s post hoc test was used. Data were obtained from the female mice. mean ± SD plotted. *P < 0.05, **P < 0.01. *n*, number of animals; HSC, hematopoietic stem cells; MDP, monocytes dendritic cells progenitor; cMoP, common monocytes progenitor cells; Mo, monocytes, MΦ, macrophages, BM, bone marrow.

We then investigated monocyte precursors in the BM compartment following hypoxia challenge. We observed evidence of active proliferation and maturation of hematopoietic stem cells into the monocyte lineage with hypoxia exposure (Figure 3A). We found a trend toward higher numbers of MDPs with a significant increase in their proliferation (Figure 3, B and C). We also observed a higher number of cMoPs with increased Ki67 expression (Figure 3, D and E). The number of classical monocytes remained consistent over time within the BM (Figure 3F), with increased Ki67 expression (Figure 3G), suggesting that these cells proliferate and depart to enter the systemic circulation. In contrast, the number of nonclassical monocytes within the BM increased only slightly over time, with no evidence of proliferation (Figure 3, H and I). Consistently, qPCR of the BM compartment showed significantly higher transcription of the classical monocyte marker *Ccr2*, with unchanged transcription of the nonclassical monocyte marker *Cx3cr1* (Supplemental Figure 11, A and B), supporting the notion that the BM increases primarily classical monocyte production during hypoxia exposure. The overall gating strategy to characterize monocytes and their progenitors in the BM is shown in Supplemental Figure 12.

### Blockade of the CCR2-CCL2 axis significantly protects from hypoxic PH.

**Figure 4:**
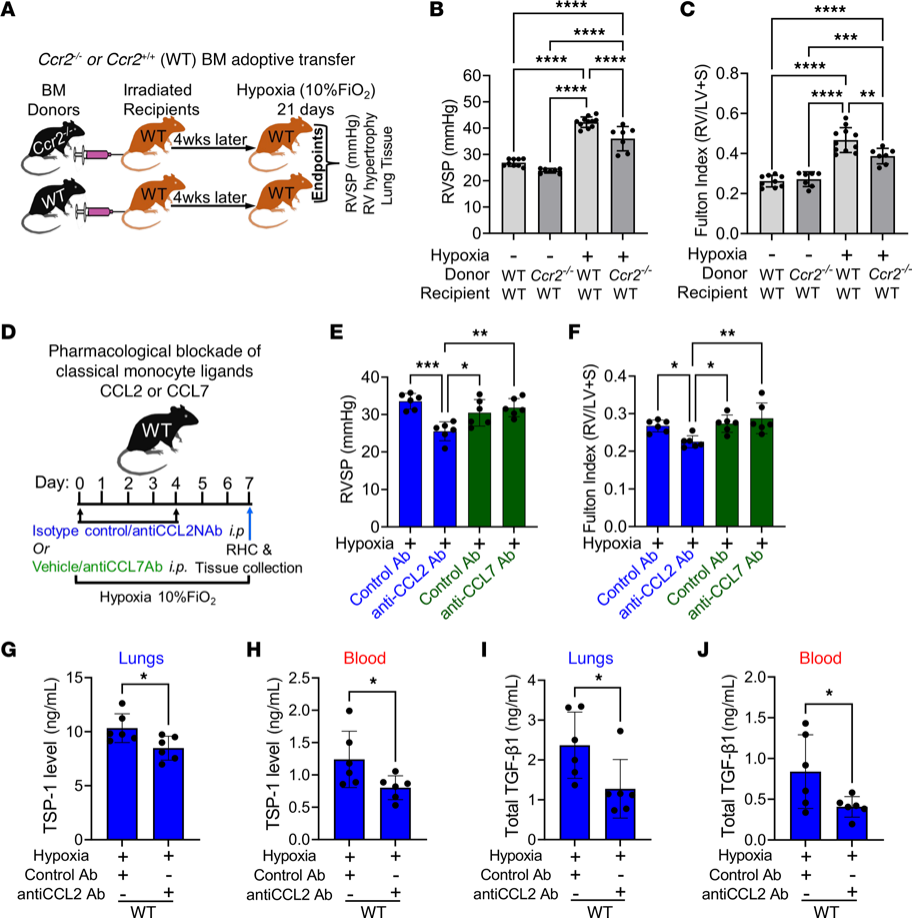
Genetic and pharmacologic blockade of CCR2-CCL2 axis protects from hypoxic PH. (**A**) Schematic showing the BM reconstitution of *Ccr2^–/–^* and WT BM into lethally irradiated WT mice. WT mice reconstituted with *Ccr2^–/–^* BM were protected from hypoxic PH by attenuated (**B**) RVSP (*n* = 7–11/group) and (**C**) RV hypertrophy (*n* = 7–11/group) as measured by Fulton Index, compared with WT mice that were reconstituted with WT BM. (**D**) Schematic showing pharmacological blockade of CCR2 ligands CCL2 or CCL7 using anti-CCL2 or anti-CCL7 neutralizing antibody treatment. Hypoxia-exposed WT mice treated with CCL2 NAb but not CCL7 NAb showed lower (**E**) RVSP (*n* = 6/group) and (**F**) RV hypertrophy (*n* = 6/group). TSP-1 levels in (**G**) lungs (*n* = 6/group) and (**H**) blood (*n* = 6/group); and TGF-β1 levels in (**I**) lungs (*n* = 6/group) and (**J**) blood (*n* = 6/group) compared with WT mice treated with isotype control antibody. Data in all panels followed a normal distribution. ANOVA with the Tukey test was performed for multiple comparisons. Data were obtained from the female mice. mean ± SD plotted. *P < 0.05, **P < 0.01, ***P < 0.001, ****P < 0.0001. *n*, number of animals; TSP-1, thrombospondin-1; TGF-β1, transforming growth factor-1; IMs, Interstitial macrophages; Ab, antibody; Nab, neutralizing antibody.

Given the evidence suggesting the CCR2-CCL2 axis may contribute to hypoxic PH, we tested its functional role. We observed, as has been reported before, that CCR2 is required for lung structural homeostasis (40, 41), as global ablation of *Ccr2* results in spontaneous PH (Supplemental Figure 13, A and B). We, therefore, specifically targeted CCR2 in the BM compartment (including classical monocytes) by transplanting *Ccr2^–/–^* BM into lethally irradiated WT mice, with WT BM transplantation into WT recipients as the control (Figure 4A). We found that WT mice that received *Ccr2^–/–^* BM cells had significantly lower RVSP (Figure 4B) and RVH (Figure 4C) following 21 days of hypoxia exposure compared with WT mice receiving WT BM. We did not observe any difference in left ventricle pressure or heart rate (Supplemental Figure 14, A–C).

We also targeted the CCR2-ligand CCL2 with an anti-CCL2 neutralizing antibody (Figure 4D), which resulted in significantly reduced RVSP and RVH following 7 days of hypoxia compared with isotype control antibody (Figure 4, E and F). We observed a reduction in the expression of TSP-1 in hypoxia-exposed lungs and blood following anti-CCL2 treatment (Figure 4, G and H), consistent with the concept that CCR2^+^ recruited IMs are a major source of TSP-1, and preventing the recruitment of CCR2^+^ IMs into the lungs confers protection by blocking TSP-1–mediated activation of TGF-β1 (Figure 4, I and J). There was no difference in left ventricle pressure or heart rate in the hypoxia-exposed WT mice that received isotype control or anti-CCL2 neutralizing antibody (Supplemental Figure 14, D-F). In contrast, anti-CCL7 neutralization did not protect against hypoxic PH (Figure 4, D–F). Of note, neither anti-CCL2 nor anti-CCL7–neutralizing antibodies affected RVSP or RV mass in normoxia (Supplemental Figure 14, G and H).

HIF2-α is a known regulator of TSP-1 (42). We analyzed TSP-1 expression in banked whole rat lungs that had been treated with a small molecule inhibitor (PT2567) that specifically blocks HIF2 activity (43, 44); it was previously reported that these PT2567-treated rats were protected from hypoxic PH (45). We found that the pharmacologic blockade of HIF2-α significantly lowered *Thbs1* expression (the gene encoding TSP-1; Supplemental Figure 15). These data collectively suggest that targeting the CCR2-CCL2 axis or CCR2-expressing classical monocytes is a potential therapeutic strategy for preventing or treating hypoxic PH.

### Resident IMs are a major source of CCL2 in hypoxic PH.

**Figure 5:**
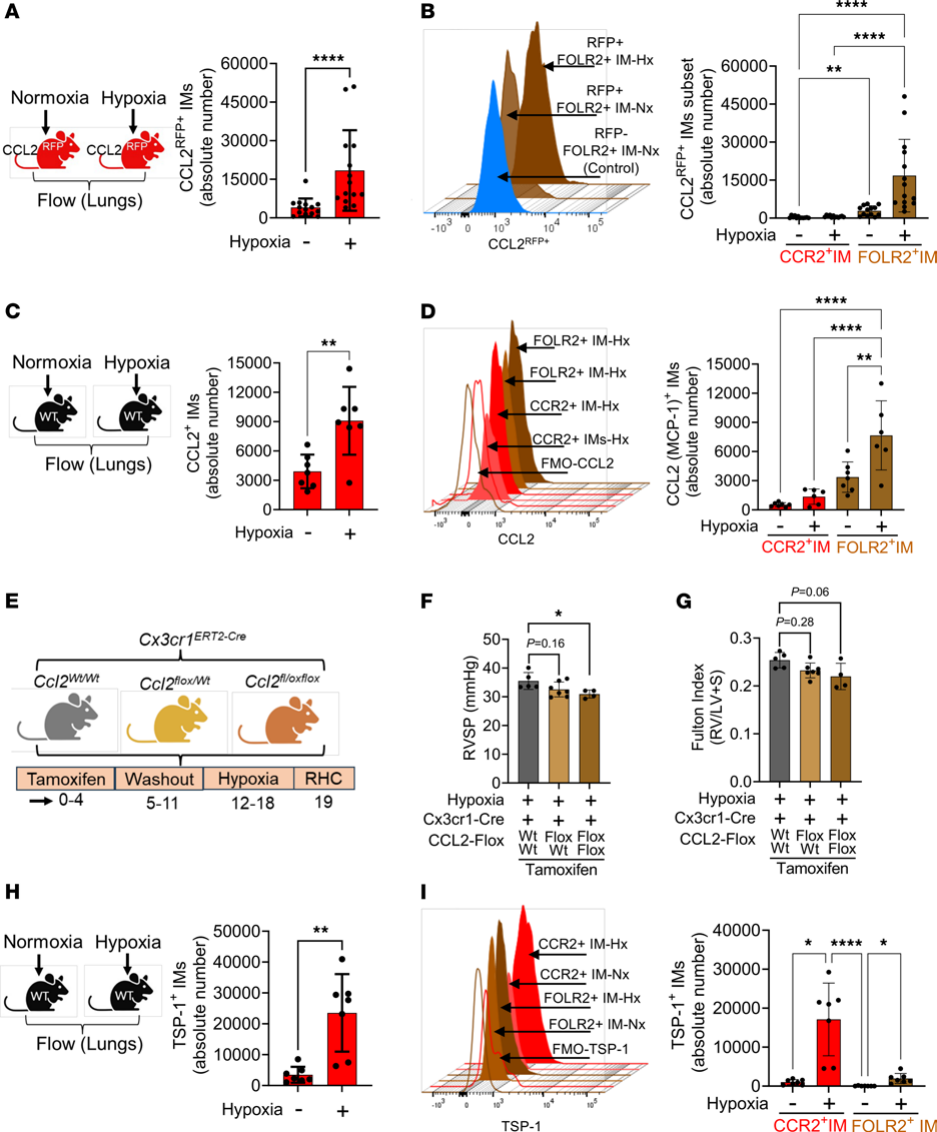
Resident IMs are a major source of CCL2 and recruited IMs are a major source of pathologic TSP-1 in hypoxic PH. (**A**) Flow cytometry analysis using *Ccl2^RFP–fl^* reporter mice showed a higher number of CCL2^+^ IMs (*n* = 14/group; *n* = 14/group, 9 female and 5 male in Nx; 8 female and 6 male in Hx), and (**B**) FOLR2^+^ IMs are a major source of CCL2 (*n* = 14/group). (**C**) Hypoxia-exposed WT mice following intracellular CCL2 staining by flow cytometry also showed a higher number of CCL2^+^ IMs (*n* = 7/group, female mice). (**D**). IM subpopulation analysis using flow cytometry also showed FOLR2^+^ resident IMs are a major source of CCL2 (*n* = 7/group, female mice). (**E**) Schematic of Tamoxifen-induced ablation of CCL2 FOLR2^+^ IMs using cre-lox system. Deletion of both copies of the *Ccl2* in Cx3cr1-expressing FOLR2 IMs showed blunted (**F**) RVSP and (**G**) Fulton Index following hypoxia exposure (*n* = 4–7/ mice group with 3 female, 2 male (WT/WT); 3 female, 4 male (WT/Fl) and 2 female, 2 male (fl/fl). (**H**) Intracellular TSP-1 staining using flow cytometry revealed recruited a higher number of TSP-1^+^ IMs (*n* = 7/group; female mice), and further analysis showed (**I**) CCR2^+^ IMs are a major source of TSP-1 in hypoxic PH (*n* = 7/group, female mice).ANOVA followed by Tukey’s post hoc test was conducted for Panels **C**, **D**, **G**, and **H**. Kruskal-Wallis ANOVA followed by Dunn’s post hoc test was used for panels **A**, **B**, **F**, and **I**. mean ± SD plotted. *P < 0.05, **P < 0.01, ***P < 0.001, ****P < 0.0001. Nx, Normoxia; Hx, hypoxia; IMs, Interstitial macrophages.

To investigate the origin of CCL2, we performed flow cytometry using *Ccl2^RFP^*-reporter mice (Figure 5A). In WT mice exposed to hypoxia, we observed a significant 1.6-fold increase in total RFP^+^ cells compared with normoxic controls (Supplemental Figure 16A). Further analysis revealed a significantly higher, 4.5-fold increase in the number of RFP^+^ IMs (Figure 5A and Supplemental Figure 16B). Specifically, RFP^+^ FOLR2^+^ IMs were significantly increased, with a 5.8-fold rise, accompanied by a significant increase in CCL2 mean fluorescence intensity (Figure 5B, Supplemental Figure 16C, and Supplemental Figure 17A). Notably, there was no increase in RFP expression in the CCR2^+^ IM subset (Figure 5B and Supplemental Figure 16D). These data were further supported by a significant increase in the number of CCL2-expressing FOLR2^+^ IMs using intracellular staining of WT mice, with increased CCL2 MFI in hypoxic compared with normoxic mice (Figure 5, C and D). While a prior study suggested that AMs are a source of CCL2 (21), we observed minimal CCL2 expression in sorted AMs and no difference in the absolute number of CCL2^+^ AMs using the *Ccl2^RFP^*-reporter mice (Supplemental Figure 17, B and C). In addition, we analyzed recently published (39) scRNA-seq on sorted IMs from normoxia and hypoxia-exposed mice and observed higher Ccl2 expression, specifically by FOLR2^+^ IMs (Supplemental Figure 17D). We observed no CCL2 upregulation in other cell types (Supplemental Figure 16, E–I). The flow cytometry gating strategy using *Ccl2^RFP^* reporter mice is shown in Supplemental Figure 18.

To understand the functional relevance of CCL2, we selectively depleted CCL2 in resident FOLR^+^ IMs by crossing CX3CR1^creERT^ with CCL2^fl^ mice. Tamoxifen was administered to delete both copies (in those with CCL2^fl/fl^ genotype), 1 copy (CCL2^fl/WT^), or no copies (CCL2^WT/WT^) of CCL2. Following tamoxifen, the mice were subjected to 7 days of hypoxia (Figure 5E). We observed that the deletion of a single copy of CCL2 resulted in a trend toward reduced RVSP, whereas the deletion of both CCL2 copies led to a significant reduction in RVSP, along with a pronounced trend toward reduced RVH compared with mice with either 1 copy deleted or both copies intact under hypoxia (Figure 5, F and G).

### Recruited IMs are a source of TSP-1 in hypoxic PH.

We utilized flow cytometry with TSP-1 intracellular staining of WT mice to identify the major cellular sources of TSP-1 in hypoxic lungs (Figure 5H). Quantitative analysis demonstrated a significant increase in the number of TSP-1^+^ cells (Supplemental Figure 19A), with a significant 6.8-fold increase in the number of TSP-1^+^ IMs in hypoxic compared with normoxic mice (Figure 5H and Supplemental Figure 19B). In hypoxia, 73% of the TSP-1^+^ IM cells were the CCR2^+^ recruited IM subpopulation (Figure 5I). With hypoxia, there were major increases in the numbers of TSP-1^+^ cells in both FOLR2^+^ and CCR2^+^ IM subpopulations (by 64- and 17-fold, respectively), but CCR2^+^ IMs likely represent the dominant TSP-1 source as they were present in larger absolute numbers than other TSP-1–expressing IMs (Supplemental Figure 19, C–I). The flow cytometry gating strategy using intracellular TSP-1 staining is shown in Supplemental Figure 20. Further analysis using the recently published scRNA-seq data (39) on sorted IMs showed higher expression of TSP-1 specifically by CCR2^+^ IMs in hypoxia (Supplemental Figure 17C), corroborating our flow cytometry findings.

### DEX prophylaxis decreases inflammatory cytokines in the peripheral blood of humans exposed to high altitude.

**Figure 6:**
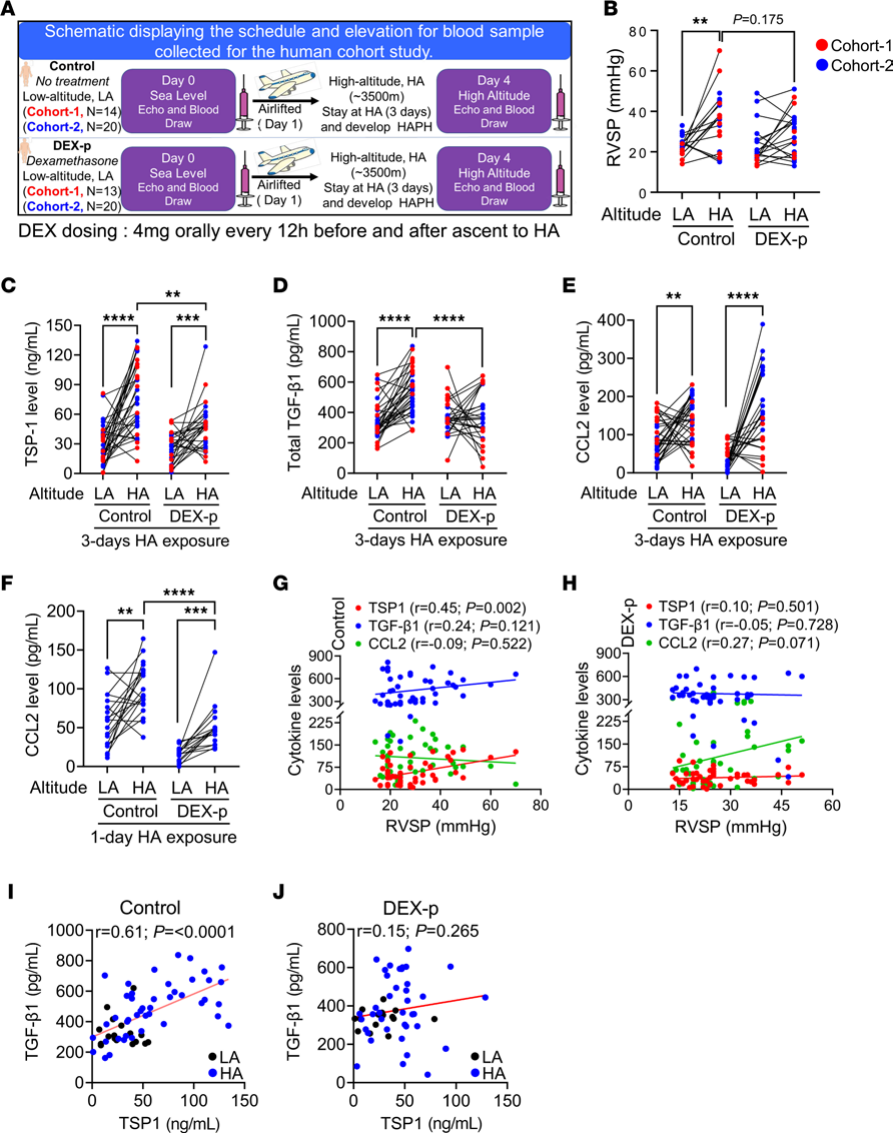
DEX prophylaxis in humans to travel to high elevation suppresses inflammatory proteins in the blood. (**A**) Schematic reviewing the study design, in which 27 individuals from cohort-1 and 40 individuals from cohort-2 were flown from LA to HA. Individuals in the control group were not treated, whereas individuals in the DEX-P group were treated with 4 mg oral twice-daily DEX prophylaxis starting 1 day prior to travel, in an unblinded manner. Blood samples were drawn before (at LA) and after 3 days of HA exposure (at HA). Samples from both cohort-1 and cohort-2 were combined and analyzed together to increase statistical power. (**B**) Paired ECHO analysis showed higher RVSP 3 days after exposure to HA, and DEX prophylactic treatment showed a mild trend toward lowering RVSP in both cohorts. This clinical data of cohort-1 was previously published (47) and is reproduced here. HA exposure resulted in higher levels of (**C**) TSP-1 and (**D**) TGF-β1, while DEX prophylaxis blunted TSP-1 (**C**) and TGF-β1 (**D**) after 3 days at HA. DEX prophylaxis showed (**E**) no effect on blood CCL2 expression at HA day 3, but (**F**) significantly lowered CCL2 at HA day 1. There was a significant correlation of (**G**) RVSP with the cytokines TSP-1 and, mildly, with TGF-β1, as well as between (**H**) TSP-1 and TGF-β1 in the untreated participants. (**I**) DEX prophylaxis abrogated the (**I**) RVSP-cytokine and (**J**) TSP-1—TGF-β1 correlations. Paired *t* tests were performed within the individuals of the same group, while unpaired *t* tests were performed between control and DEX-p HA-exposed individuals. **P < 0.01, ***P < 0.001, ****P < 0.0001.

DEX prophylaxis is used to prevent development of HA illnesses (31, 46). Despite its known antiinflammatory properties, it is unclear how DEX works in this setting, and therefore provides an opportunity to explore how inflammation modulation may impact HA-induced pulmonary diseases such as PH. We hypothesized that prophylactic DEX may confer protection by inhibiting the CCR2-CCL2 axis, thereby preventing the recruitment of TSP-1^+^ IMs. We evaluated peripheral blood signatures in banked specimens from a previously published cohort-1 study (47) and another new cohort-2 study, in which individuals traveled from low altitude (LA; Delhi, India, with an altitude of 225 meters above sea level) to high altitude (HA; Ladakh, India, 3,500 meters above sea level), where they remained for 3 days (the cohort-2 data is presented in Figure 6A). Fourteen individuals in cohort-1 and 20 individuals in cohort-2 received no treatment (not blinded; control group), while 13 individuals in cohort-1 and 20 individuals in cohort-2 prophylactically received oral 4 mg DEX twice daily starting 1 day before traveling to the HA (DEX-p group). RVSP was estimated by echocardiography. The combined clinical data of cohort-1 (47) and cohort-2 (Figure 6B) suggest that DEX prophylaxis may mildly mitigate acute high altitude PH (HAPH), a type of hypoxic PH, as the DEX-p group at HA had a nonsignificant trend toward lower RVSP (post hoc *P* values when accounting for multiple comparisons: *P* = 0.175).

In the control group of both cohort-1 and cohort-2 that did not receive DEX, plasma TSP-1, total TGF-β1 and CCL2 levels significantly increased after 3 days of HA exposure, with 2.5, 1.6, and 1.5-fold changes, respectively (Figure 6, C–E). Notably, the DEX-p group at HA had significantly lower plasma TSP-1 and total TGF-β1 (1.6 and 1.4-fold reductions, respectively) compared with the control group at HA (Figure 6, C and D). Surprisingly, DEX prophylaxis did not attenuate CCL2 levels with the HA exposure (Figure 6E). To investigate if DEX has an early response in CCL2 blockade in humans, we measured CCL2 levels in cohort-2 samples after 1 day of HA exposure (early samples from cohort-1 were not available). Interestingly, we observed a significant 1.7-fold increase in CCL2 in untreated individuals while DEX prophylaxis significantly attenuated by 2-fold the CCL2 level after 1 day of HA exposure (Figure 6F). In control individuals, there was significant correlation between RVSP and TSP-1 (*P* = 0.002; Figure 6G), which was lost among DEX-p treated individuals (Figure 6H). Similarly, there was a strong collection between TGF-β1 and TSP-1 among controls (*P* < 0.0001), which was lost among the DEX-p individuals (Figure 6, I and J). The previously published clinical study found that DEX prophylaxis resulted in a greater level of protection in females than in males (47). We explored whether DEX prophylaxis had sexually dimorphic effects on RVSP and inflammatory cytokines. We observed the highest RVSP, TSP-1, and TGF-β1 levels in untreated, control female participants at HA, which was effectively suppressed by DEX prophylaxis (Supplemental Figure 21, A–C). We did not observe any sexual dimorphism in CCL2 protein concentrations (Supplemental Figure 21D).

### DEX prophylaxis in the preclinical hypoxic model recapitulates human findings by blocking inflammatory cytokines and protecting from acute PH.

**Figure 7:**
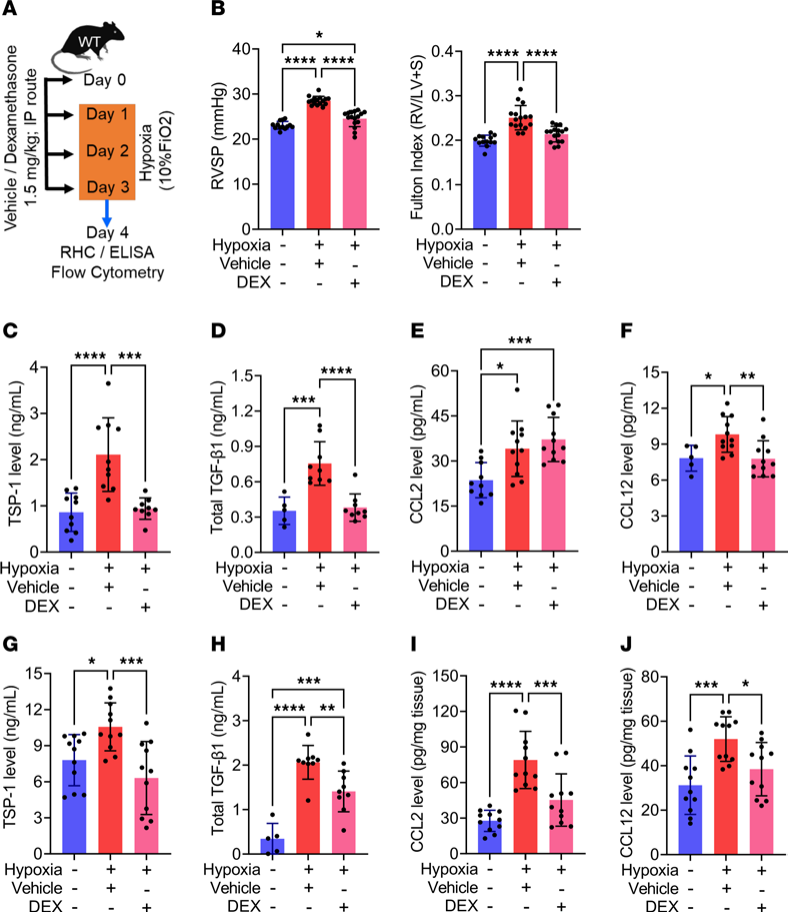
DEX prophylaxis protects from hypoxic PH by blocking inflammatory cytokines both in the lungs and in circulation. (**A**) Schematic showing the experimental design and the dosing of DEX prophylaxis. Hypoxia-exposed WT mice treated with DEX prophylaxis had lower (**B**) RVSP and RVH by RHC (*n* = 13–16/group). The blood (**C**) TSP-1 (*n* = 5–11/group) and (**D**) TGF-β1 (*n* = 5–9/group) levels were blunted by DEX prophylaxis treatment in hypoxic PH. The blood monocytes ligands (**E**) CCL2 (*n* = 11/group) was significantly upregulated whereas (**F**) CCL12 (*n* = 11/group) was downregulated following hypoxia exposure in the DEX prophylactically treated group. The levels of inflammatory proteins (**G**) TSP-1 (*n* = 11/group) and (**H**) TGF-β1 (*n* = 5–9/group) were significantly lower in the lung WT mice treated with DEX prophylaxis. DEX prophylaxis blunted classical monocyte ligands (**I**) CCL2 (*n* = 11/group) and (**J**) CCL12 (*n* = 11/group) in lung tissue lysates by ELISA. Data in all panels were obtained from female mice and followed a normal distribution. Statistical analysis was conducted using ANOVA, followed by Tukey’s post hoc test. mean ± SD plotted. *P < 0.05, **P < 0.01, ***P < 0.001, ****P < 0.0001. RVSP, right ventricular systolic pressure; RV, right ventricle; IMs, interstitial macrophages; DEX, Dexamethasone.

To investigate if hypoxia-exposed mice can recapitulate the human condition at HA and serve as an experimental model for investigating mechanisms by which DEX may function in this context, mice were given DEX, at a dose equivalent to human dosing (31, 46, 47), starting 1 day prior to hypoxia. We observed that DEX prophylaxis significantly reduced RVSP and RVH in WT mice challenged with 3 days of normobaric hypoxia, compared with vehicle-treated controls (Figure 7, A and B). To corroborate these data at normobaric hypoxic conditions, and to mimic human exposure to HA, we also used a hypobaric chamber equivalent to 18,000 ft (approximately 5,500 m) elevation, and observed DEX attenuated the rise in RVSP and RVH in WT mice exposed in this manner (Supplemental Figure 22, A–C). Similar to the human observations, DEX-mediated protection during hypoxia exposure was accompanied by decreased TSP-1 and TGF-β1 levels in the blood (Figure 7, C and D). We found the elevated levels of CCL2 in the blood were interestingly not decreased by DEX prophylaxis (Figure 7E), whereas CCL12 levels were abrogated (Figure 7F) compared with vehicle-treated mice following hypoxia exposure. We also analyzed the lungs from these mice and observed that the protection was accompanied by decreased expression of TSP-1, TGF-β1, CCL2, and CCL12 in DEX prophylaxis–treated compared with vehicle-treated mice following hypoxia exposure (Figure 7, G–J). We also analyzed the long-term efficacy of DEX in protecting from more chronic PH utilizing a mouse model of 21 days hypoxia exposure and observed a significant reduction in the RVSP and RVH for the mice on DEX prophylaxis compared with vehicle (Supplemental Figure 23, A–C).

We previously observed that TGF-β activation in HAPH causes vasoconstriction by ρA/ρ kinase (ROCK) signaling (8). We therefore explored if DEX suppresses ROCK signaling. We observed that acute intravenous administration of fasudil, a potent ROCK inhibitor, significantly reduced RVSP in hypoxia-exposed WT mice, with a more pronounced effect in females than males (Supplemental Figure 24, A and B). In DEX-treated hypoxia-exposed female mice, fasudil still lowered RVSP (although significantly less than in vehicle-treated hypoxia-exposed animals), whereas fasudil did not further reduce RVSP in DEX-treated male mice. These data suggest that DEX treatment significantly but not completely suppressed ROCK activation.

### DEX prophylaxis blocks the recruitment of TSP^+^ IMs by abrogating the CCL2 gradient between blood and lungs and protects from preclinical hypoxic disease.

**Figure 8:**
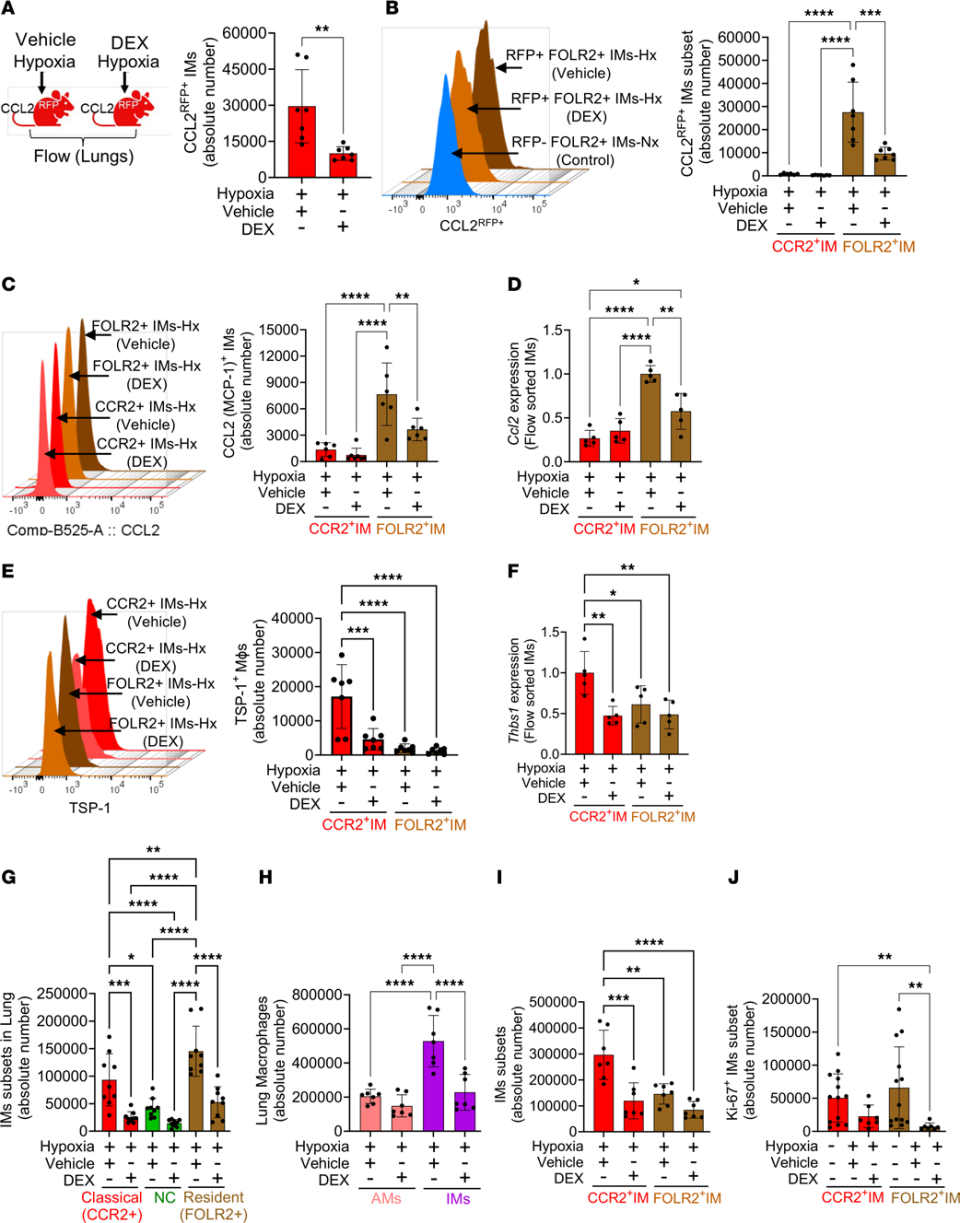
DEX prophylaxis blunts CCL2 production by resident IMs and blocks the recruitment of TSP-1–producing CCR2^+^ IMs in hypoxia. (**A**) DEX prophylactically treated, hypoxia-exposed *Ccl2^RFP–fl^* reporter mice exhibited a significant reduction in CCL2^+^ IMs, particularly in (**B**) CCL2^RFP+^ resident IMs (*n* = 7/group). Additionally, (**C**) intracellular CCL2 flow cytometry analysis in DEX prophylactically treated hypoxia-exposed WT mice revealed a decrease in CCL2 expressing FOLR2^+^ resident IMs (*n* = 7/group, female mice). (**D**) qPCR on flow-sorted FOLR^+^ IM from DEX-prophylactically treated hypoxia-exposed WT mice showed lower *Ccl2* expression. (**E**) DEX prophylaxis attenuated the TSP-1 expressing CCR2^+^ IMs in hypoxia (*n* = 7/group, female mice). (**F**) qPCR on flow-sorted CCR2^+^ IM from DEX prophylactically treated hypoxia-exposed WT mice showed lower *Thbs1* expression (*n* = 5/group, female mice). (**G**) Double-reporter mice displayed a marked reduction in RFP^+^ IMs and resident IMs in the hypoxia-exposed DEX prophylatically treated group (*n* = 9/group; 4 female, 5 male in vehicle; 5 female, 4 male in DEX group). Moreover, DEX prophylaxis further decreased (**H**) total IMs (*n* = 7/group, female mice) by abrogating the recruitment of (**I**) CCR2^+^ IMs and reducing the number of FOLR2^+^ IMs (*n* = 7/group, female mice) via (**J**) blocking proliferation, as indicated by Ki-67 expression (*n* = 7–13/group, female mice). Statistical analysis was conducted using ANOVA, followed by Tukey’s post hoc test for all the panels except panel **J**. Kruskal-Wallis ANOVA followed by Dunn’s post hoc test was used for panel **J**. mean ± SD plotted. *P < 0.05, **P < 0.01, ***P < 0.001, ****P < 0.0001.

We interrogated the impact of DEX on CCL2 expression using the *Ccl2^RFP^* reporter mice. DEX has been reported to block CCL2 expression by microglia (48). We observed that DEX prophylaxis blunted CCL2 expression by the lung IMs following hypoxia exposure (Figure 8A), specifically in the FOLR2^+^ resident IM subpopulation (Figure 8B and Supplemental Figure 17A). These data were further supported by decreased intracellular staining of CCL2 in the FOLR2^+^ resident IMs in WT mice following DEX prophylaxis (Figure 8C). Further, to confirm the flow cytometry data, we flow-sorted lung CCR2^+^ and FOLR2^+^ IMs from hypoxia-exposed WT mice with and without DEX-prophylaxis and observed a significant reduction in *Ccl2* mRNA expression in FOLR2^+^ IMs in the DEX-prophylaxis group (Figure 8D). Consistent with a disrupted CCL2 gradient, DEX prophylaxis abrogated recruitment of TSP-1^+^ expressing CCR2^+^ IMs in hypoxia-exposed lungs (Figure 8E). Furthermore, we observed reduced *Thbs1* mRNA expression in flow-sorted CCR2^+^ IMs (Figure 8F). These data thus corroborate the observed reduction in TSP-1 and TGF-β1 levels in the blood (Figure 7, C and D) and the lungs (Figure 7, G and H) with DEX prophylaxis, and also suggest that TSP-1 expression may directly be suppressed, at least in part, by DEX treatment. Furthermore, prophylactically DEX-treated *Ccr2^+/RFP^Cx3cr1^+/GFP^* reporter mice demonstrated fewer RFP^+^ classical monocyte precursors in the lungs (Figure 8G). We further investigated the effect of DEX prophylaxis in the intravascular compartment. We noted significantly more RFP^+^ classical monocyte precursors in the intravascular compartment of DEX-treated double-reporter mice (Supplemental Figure 25A). These intravascular precursor classical monocytes had higher TSP-1 expression in DEX-treated WT mice (Supplemental Figure 25B). These data were complemented by DEX prophylaxis in WT mice resulting in fewer lung IMs (Figure 8H). IM subtype analysis revealed attenuation in both CCR2^+^ recruited IMs and FOLR2^+^ resident IMs (Figure 8I). DEX prophylaxis also significantly attenuated the proliferation of FOLR2^+^ resident IMs following hypoxia exposure (Figure 8J). Collectively, these data are consistent with the concept that DEX prophylaxis suppresses TSP-1 production in the lungs by disrupting the CCL2 gradient between the lungs and blood, trapping classical monocytes in the intravascular compartment and preventing recruitment of inflammatory CCR2^+^ TSP-1-expressing monocytes into the lungs.

Lastly, we investigated if monocyte egress from the BM shares CCL2-mediated signaling. Blockade of CCL2 using anti-CCL2 NAb attenuated the total number of IMs in the lungs, with a reduction in both CCR2^+^ and FOLR2^+^ subsets, and reduced proliferation of FOLR2^+^ IMs (Supplemental Figure 26, A–C). CCL2 blockade also resulted in fewer circulating classical monocytes in the blood (Supplemental Figure 26, D–F). However, we observed no significant differences, and, in fact, mild trends toward increased monocyte and monocyte progenitors in the BM compartment (Supplemental Figure 26, G–J). These data overall suggest that the CCL2 gradient emanating predominantly from the lungs is required for the egress and recruitment of monocytes into the intravascular and lung compartment, respectively, but does not directly drive hypoxia-driven BM changes.

## Discussion

Inflammation in the lungs and pulmonary vessels is recognized as a hallmark of multiple forms of human PH (49–51) and experimental PH in animal models (52–55). Hypoxia triggers early and persistent pulmonary artery–specific inflammatory responses, including mononuclear cell recruitment (2, 8, 56), resulting in vascular injury and remodeling (2, 57). The severity of PH correlates with the degree of inflammation (58). In experimental models, antiinflammatory treatments including glucocorticoids (32, 59); blocking TGF-β (60–62), IL-6 (63, 64), IL-21 (64), or SDF-1 (65); and inhibiting macrophages (64, 66) all attenuate PH. Despite the large body of data linking inflammation with PH, systemic antiinflammatory strategies have not clinically shown a clear benefit in patients with PH, with rare exceptions, as is seen in lupus-associated PH (67–69). This hindrance to clinical translation underscores the currently incomplete mechanistic understanding of how inflammation contributes to PH.

By using multiple orthogonal approaches, we found that hypoxia leads to CCL2 production by resident FOLR2^+^ IMs, which contributes to the recruitment of CCR2^+^ classical monocytes that serve as precursors of recruited TSP-1^+^ IMs. This inflammatory cascade results in the development of hypoxic PH through TSP-1-mediated activation of TGF-β1. Additionally, we found that DEX prophylaxis blocks these pathological phenotypes in both humans and mice, revealing a molecular mechanism by which corticosteroids may aid acute HA illnesses (Supplemental Figure 27).

These results demonstrate the involvement of classical monocytes at early time points, whereas others have studied nonclassical monocytes at later time points (10, 70); how the 2 subpopulations are related is unclear. Studies in other pulmonary conditions have also shown that classical monocytes can function as the precursor of inflammatory macrophages in the lungs (17). The BM is the source of monocytes derived from cMoPs (13), which are released to the circulation through CCL2/CCL12 (15, 18, 71) signaling. Our observations here corroborate previous findings that depleting circulatory monocytes with clodronate or blocking monocyte receptors reduces recruitment of blood monocytes into lung perivascular spaces and their subsequent differentiation into inflammatory IMs (8, 9).

Prior studies have indicated that depleting the classical monocyte receptor CCR2 or its ligand CCL2 globally have detrimental effects on organ structure and/or function (40, 70). Mice lacking CCL2 display comparable phenotypes with those lacking CCR2, suggesting that the CCL2-CCR2 pairing is a relevant interaction in vivo (72, 73). To avoid baseline confounding phenotypes in CCR2-CCL2 biology in hypoxic PH, we used a *Ccr2^–/–^* BM chimera approach to block CCR2 in BM cells. This approach and the blockade of CCL2 using a neutralizing antibody partially protected WT mice from hypoxic PH.

Our flow cytometry data using a *Ccl2^RFP^* reporter and intracellular CCL2 expression in WT mice indicate that IMs are an important cellular source of CCL2, and specifically the resident FOLR2^+^ IM subpopulation (11, 12), which may directly sense hypoxia and produce CCL2. Prior studies have suggested that AMs can also produce CCL2 (21); however, in line with another report (74), we observed minimal CCL2 expression in AMs. These observations suggest a pathologic interplay between 2 key IM subpopulations: (a) resident FOLR2^+^ IMs producing CCL2, which promote the recruitment of (b) CCR2^+^ IMs expressing TSP-1. Of note, based on our data, we observed greater proliferation among FOLR2^+^ resident IMs, which corroborates the recent discovery that these cells maintain their population through self renewal (12).

Further, we tested if DEX prophylactic treatment can blunt CCR2-CCL2–driven inflammation. Using a similar dose to that used to prevent HA illness in humans (31, 46, 47), we observed that mice prophylactically treated with DEX had higher CCL2 levels in the circulation and lower CCL2 levels in the lungs, and lower CCL12 levels in both compartments, which aligned with retention of classical monocytes in the intravascular compartment. DEX prophylaxis thus appears to block monocyte recruitment into the lungs by disrupting the lung-blood CCL2 gradient, which is necessary for classical monocytes to enter the lung parenchyma (75, 76). This block resulted in reduced TSP-1^+^ IM recruitment, with less TSP-1 and TGF-β1 expression in the lungs. DEX treatment has been used in the monocrotaline-PH model, resulting in reduced vascular smooth muscle cell proliferation (32, 33), but these studies used an equivalent steroid dose much higher than clinically used doses (77, 78) and lacked clear mechanisms.

Recent work has found that HIF-1α induces CCL2 (79, 80), whereas HIF-2a induces TSP-1 (42). Our findings here suggest a model of cell compartment–specific and HIF isoform–specific regulation, wherein HIF-1α stabilization under hypoxia triggers CCL2 production, which subsequently signals to intravascular monocytes, leading to their recruitment into the lungs. In the hypoxic lung environment, HIF-2α is stabilized in these recruited pre-IM precursor monocytes, leading to TSP-1 production. These hypothesized macrophage subset-specific, HIF isoform–specific roles still need to be experimentally tested.

To understand the potential clinical implication of our research, we examined the effect of DEX prophylaxis in banked samples from people who had traveled from LA to HA without acclimatization. We observed increased plasma TSP-1 and TGF-β1 in participants in the control group who were exposed to HA. In both cohorts, we observed that DEX prophylaxis was associated with reduced TSP-1 and TGF-β1 and a modest decrease in CCL2 — similar findings to our observations in the mice. These data are consistent with the concept of a disrupted lung-blood CCL2 gradient, which is required to facilitate the migration of classical monocytes into the lung tissue through a combination of suppressed lung CCL2 expression and blood CCL2 expression, which may be decreased (at day 1) or relatively unchanged (by day 3). The difference in phenotype between the mouse model and humans may be due in part to a milder stimulus in the human participants, as 3,500 m altitude has a partial pressure of oxygen (pO_2_) equivalent to 13.5% fraction of inspired oxygen (F_i_O_2_). Furthermore, we observed that TSP-1 mRNA expression by recruited IMs may be partially suppressed by DEX treatment, potentially independent of a CCL2-driven mechanism.

Additional considerations may impact the overall interpretation of our data. Although we found that FOLR2^+^ IMs are a source of CCL2 in hypoxia, it is unclear if FOLR2^+^ IMs in other organs may also produce CCL2 upon hypoxia exposure. Moreover, other cell types may also contribute to CCL2 production in hypoxia. We observed the heart and liver also produce CCL2 in hypoxia, but significantly less than the lungs. Hypoxia-induced phenotypes represent specific cellular programming that appears unique for each organ. The pulmonary expression of CCL2 may represent an innate adaptive immune response relatively specific to this barrier organ that is frequently exposed to infectious and noninfectious stimuli. Future studies studying the expression of CCL2 and its analogs in the context of different stimuli, and if it is retained from lower lifeforms, may help answer these important questions.

We also observed a trend toward increased CCL2 expression by other stromal cell types, which likely contributes to monocyte recruitment, and targeting CCL2 expression by FOLR2^+^ IMs alone may not block the monocyte recruitment cascade. It is unclear if HIF stabilization or other mechanisms contribute to the expression of CCL2 by FOLR2^+^ IMs directly. In mice, CCL12 may also be contributory (although there is no biologic equivalent of CCL12 in humans), and we have not tested its potential function. We found that blocking CCR2-CCL2 partially abrogated the phenotype, suggesting that other parallel mechanisms are also contributing to hypoxic PH; the combination of blocking CCR2 and its ligands may enhance the impact. DEX prophylaxis may have other effects, such as blocking TSP-1 or TGF-β expression, independent of its observed impact on monocyte recruitment.

Overall, this study suggests that hypoxia triggers a cell-specific role of CCR2—CCL2 signaling in the lungs, which contributes to the development of PH following exposure to hypoxia. Therapeutics that specifically target the CCL2/CCR2 chemokine axis are currently in clinical trials for cancer indications (81). This study also suggests mechanisms by which DEX prophylaxis may mitigate HA illnesses in humans.

## Methods

### Sex as a biological variable.

Our study examined both males and females in experimental models and human biospecimen studies, including differences between male and female mice and humans.

### Animals.

WT (C57BL6/J; stock # 000664), *Ccr2^RFP^Cx3cr1^GFP^* dual-reporter mice (Stock # 032127), *Ccl2^RFP–fl^* (stock # 016849), and *Ccr2^–/–^* (stock # 004999); *R26Stop^(fl/fl)tdTomato^* (stock # 007909); *Cx3cr1^CreER^* (stock # 020940); *Ccr2^CreER–GFP^* (stock # 035229) mice were purchased from Jackson Laboratories. Six-to-eight week-old mice were used for the experiments. All animals were kept under specific pathogen-free conditions in an American Association for the Accreditation of Laboratory Animal Care–approved facility at the University of California San Francisco (UCSF) or the University of Colorado Anschutz Medical Campus (CUAMC). Experiments were performed in a blinded format. Mice were euthanized by sedation with ketamine-xylazine, and bilateral thoracotomy followed by exsanguination was performed. cDNA samples from Dawley male rats treated with a small molecule inhibitor (PT2567; Peloton Therapeutics) that specifically blocks HIF2 activity or vehicle (0.5% methylcellulose/0.5% Tween-80), were provided by Kurt Stenmark (University of Colorado Anschutz Medical Campus). The hemodynamic data on these rats have been recently published (45).

### Hypoxia exposure of mice.

Experimental mice were exposed to 10% fraction of inspired oxygen (F_i_O_2_) in a normobaric hypoxia chamber for 1, 3, or 21 days to perform time course–based hemodynamic measurements and flow cytometry analysis as has been done previously (7, 8). Mice were placed in the hypoxia chamber connected to the oxygen feedback sensor ProOx 360 (BioSpherix) that senses and maintains the target oxygen concentration inside the chamber by infusing nitrogen (N_2_) gas, which is mixed using a fan. Mice in room air served as normoxic controls. We also used a chamber equipped for continuous hypobaric hypoxia (corresponding to an altitude of approximately 5500 m) to expose WT mice. Matched WT control mice were housed in identical chambers at sea level atmosphere. Mice underwent terminal right heart catheterization and/or flow cytometry analysis on collected lung tissues, PBMCs, and BM cells. Lung tissues and plasma samples were used to perform RNA and protein assessments.

### Hemodynamics measurement.

Measurement of right ventricular systolic pressure (RVSP) and right ventricle hypertrophy (RVH) was performed as previously described (7, 8). Briefly, mice were sedated with ketamine/xylazine and ventilated through a transtracheal catheter. Abdominal and thoracic cavities were opened and a 1Fr pressure-volume catheter (PVR-1035, Millar Instruments) was placed through the right ventricle apex to transduce the pressure in the right ventricle cavity, followed by measuring the pressure in the left ventricle cavity.

### Protein Quantification by ELISA.

Commercial ELISA kits were used for the quantification of CCL2 (Cat.#MJE00B, R&D Systems), CCL7 (Cat.# MBS764604, MyBioSource), CCL12 (Cat.#MCC120, R&D Systems), CXCL1 (Cat.#MCX310, R&D Systems), TSP-1 (Cat.# MBS264008, MyBioSource), and TGF-β1 (Cat.#sMB100B and DB100C, R&D Systems) using whole lung lysates and plasma samples from unexposed and hypoxia-exposed mice. The assays were done per the manufacturer’s instructions in duplicate. To measure total TGF-β1, samples were HCl activated, per protocol.

### Bone marrow cell analysis.

Bone marrow (BM) cells were harvested per the previously published protocol (82). Skins were removed from both legs of experimental mice with scissors without compromising the BM cavity. The tibia and femur were removed and placed in RPMI media containing 10% FBS. BM cells were flushed from the tibia and femur using 2–3 mL of media through 27-gauge needle. The cells were then passed through a 100 μm strainer, centrifuged, and RBCs lysed. The cells were counted using hematocytometer. Flow cytometry on BM cells was performed after staining with antibodies as described below.

### RNA quantitation.

To quantify mRNA transcript expression of *Ccr2* and *Cx3cr1* in the BM compartment, we used real-time polymerase chain reaction (RT-PCR) with primer information listed in Supplemental Table 1. First-strand cDNA synthesis was conducted with the iScript cDNA Synthesis kit (Bio-Rad Laboratories) using 1 μg RNA. Each gene and sample were analyzed in duplicate using RT-PCR. TaqMan Gene Expression Master Mix (Applied Biosystems) was used to assess the expression of target genes on the StepOnePlus Real-Time PCR System (Applied Biosystems). The reaction volume was 20 μL. To determine relative transcript quantities, we used the 2^–ΔCt^ method with β-actin as the reference gene.

### Bone Marrow Transplantation.

Eight-week-old WT (C57BL/6) and *Ccr2^–/–^* (C57BL/6 background) mice were used as bone marrow donors, and 6-week-old WT mice were used as recipients for bone marrow transplantation (BMT). Recipient mice were irradiated with 10 Gy, split into 2 fractions 4 hours apart using a cesium irradiator. Purified donor cells (2–4 × 10^6^) were injected intravenously. BM chimera mice were kept on trimethoprim-containing chow for 4 weeks. One week after stopping the trimethoprim-containing chow, the mice were exposed to hypoxia. Controls were kept at room air.

### Neutralizing antibody and pharmacological treatment.

Neutralizing mouse antibodies against CCL2 (Clone: 2H5; Cat.# BE0185), CCL7 (R&D System; Cat.# AF-456-NA), and isotype control (Cat.# BE0091; BioXCell) were reconstituted in phosphate-buffered saline (PBS). Both CCL2 (200 μg/mouse) and CCL7 (20 μg/mouse) neutralizing antibodies were injected i.p. just before placing the mice in a hypoxia chamber (Biospherix), which was repeated every 4 days. The dose quantity and treatment schedule of both neutralizing antibody treatments were based on prior reports (77, 83). For DEX prophylaxis in mice, 1.5 mg/kg of DEX (Cat.# D2915, Sigma-Aldrich) was administered i.p. once daily, starting 1 day prior to hypoxia exposure and continuing until the conclusion of the experiment.

### PBMC and plasma isolation.

PBMCs were harvested from mice using the Ficoll gradient method. Briefly, 300–400 μL blood was drawn in EDTA-containing tubes. An equal volume of PBS was then added to the blood mixture, which was carefully layered over Ficoll solution in tubes (Cat.#17-144-02, GE Healthcare). Subsequently, the tubes were centrifuged at 400*g* at room temperature for 30 minutes. The buffy coat layer was removed from each tube and washed with PBS. For protein quantification, plasma was isolated from EDTA tubes containing freshly isolated blood, centrifuged at 400*g* for 20 minutes at room temperature.

### Mouse lung digestion.

Unexposed and hypoxia-exposed mice were sedated with ketamine/xylazine, and the lungs perfused with PBS to remove circulating cells, followed by harvest and digestion to make a single-cell suspension, as has been done previously (8). Briefly, perfused lungs were digested using liberase (Roche) dissolved in RPMI (Mediatech) at a concentration of 1 mg/mL at 37°C for 30 minutes. The digested tissue was further disrupted by passing through 16 G (5×) and 18 G (5×) needles, sequentially. The cells were filtered using a 100 μm cell strainer (Thermo Fisher Scientific) and the cell pellets collected after centrifugation for 5 minutes at 300g. Red blood cells were lysed using 1 mL of ACK lysis buffer (Gibco). The remaining cells were resuspended and washed in RPMI to neutralize the lysis buffer. Single-cell suspensions were further filtered and collected into flow wash buffer (5% BSA in PBS with EDTA) for flow cytometry staining.

### Flow cytometry.

To label circulating cells, mice were retroorbitally injected with an AF700-labeled anti-CD45 antibody (1 μg/mouse) 5 minutes prior to euthanasia. The digested single-cell suspension (5–10 × 10^6^ cells) was preincubated with a CD16/CD32 antibody (eBioscience) for 20 minutes to block nonspecific Fc-γ receptor-mediated antibody binding. The cells were then stained with appropriate fluorochrome-conjugated antibodies at 4°C in the dark for 30 minutes. For nuclear Ki67 and intracellular TSP-1 and CCL2 staining, extracellular-stained cells were fixed and permeabilized first using Foxp3 fixation/permeabilization buffer (eBioscience, Cat# 00-5523-00) and stained using appropriate antibodies. Details of the clone and antibody concentrations are in Supplemental Table 2. Cell viability was assessed using LIVE/DEAD Fixable Viability Dye eFluor 450 violet stain (eBioscience, Cat# 65-0863-14) and the cell suspensions were analyzed with an LSRII (BD Biosciences). Results were analyzed using FlowJo software (v-10.9.0). The gating strategy was similar to our prior report (8). Briefly, cells were sorted after gating singlets, live (viability dye^−^), and lineage-negative cells (T cells, B cells, NK cells, and neutrophils). The surface markers used to classify mouse immune cell types in the BM, blood, and lung tissue are listed in Supplemental Table 3.

### Alveolar macrophage isolation.

BAL fluid was collected using instillation of 1 mL PBS through the tracheostomy. The collected fluid was centrifuged (400*g* for 10 minutes at 4°C), and the cell pellet was washed with 1× PBS. The pellets were further incubated for 20 minutes with antibodies against the macrophage marker F4/80, labeled with magnetic beads; each mixture was then passed through a positive-selection magnetic column (Miltenyi). After washing the column with 10 mL of column wash buffer, AMs were collected using a plunger. The cells were counted using a cell counter, and viability (greater-than 90%) was checked using trypan blue dye.

### IM flow sorting.

To sort CCR2^+^ and FOLR2^+^ IM subpopulations, we used single-cell suspension. The cells were labeled with antibodies as describes above. Sorting was done using a BD FACSAria III Cell Sorter. RNAs were extracted from the AMs and sorted IMs using Qiagen mini RNA isolation kit (Cat.# 74004), and RNA concentration quantified using NanoDrop. A total of 0.3μg of RNA from each sample was used for reverse transcription and *Ccl2* mRNA quantification by RT-PCR (see RNA quantitation above).

### Human biospecimens.

Banked samples from a previously conducted and published study were analyzed (47). This study included 2 cohorts comprised of 67 age and sex-matched healthy participants recruited in collaboration with the Council of Scientific and Industrial Research (CSIR) – Institute of Genomics and Integrative Biology, Delhi, India, and the Sonam Norboo Memorial (SNM) Hospital, Leh, Ladakh, India. Individuals with chronic diseases, pulmonary infection (including SARS-CoV-2 infection), pregnant status, or who were unable to give informed consent were excluded from the study. The participants were divided into 2 groups in an unblinded manner: control group and dex-p group. In cohort-1, 14 individuals and in cohort-2, 20 individuals received no treatment (Control). Participants in the DEX-p group comprised 13 individuals in cohort-1 and 20 individuals in cohort-2, who received prophylactic oral DEX treatment (4 mg twice a day; Wockhardt Ltd.) under the supervision of clinical investigators. Standard clinical parameters were first measured at the LA, (Delhi, India, 225m), then again 3 days after the individuals were flown to and remained at the HA (Ladakh, India, 3500m). In the DEX-p group, DEX treatment was started 1 day prior to travel and continued for the study duration. Ultimately, blood samples were drawn from 32 participants in the control group and 27 participants in the DEX-p group, both at LA and HA, generating 4 groups of blood samples. Quantification of CCL2, TSP-1, and TGF-β1 levels were performed on the plasma samples using the commercial ELISA kits (Cat.# SEA087Hu, SEA611Hu, and SEA124Hu, respectively, from Cloud-Clone Corp). The assays were done per the manufacturer’s instructions in duplicates.

### Statistics.

All results are reported as mean ± SD. The Shapiro-Wilk test was used to assess whether the data were normally distributed. Differences between 2 groups were analyzed using an unpaired *t* test, while differences among 3 or more groups were assessed using ANOVA followed by Tukey’s post hoc test. A paired 2-tailed *t* test was used for comparisons between human samples taken at 2 time points (LA versus HA). For nonnormally distributed data, the Mann-Whitney U test was used for comparisons between 2 groups, and the Kruskal-Wallis test, followed by Dunn’s post hoc test, was applied for comparisons among more than 2 groups. *P* values less than 0.05 were considered statistically significant. Prism (v9, GraphPad) was used for statistical analyses and graphs.

### Study approval.

All experimental protocols using animal studies were approved by the UCSF and CUAMC Institutional Animal Care and Use Committees. The human study protocol was approved by the human ethical committees of the CSIR-Institute of Genomics and Integrative Biology, Delhi, India, and by the SNM Hospital, Leh, Ladakh, India. All procedures were performed in compliance with relevant laws and institutional guidelines. Informed consent was obtained from each individual.

### Data availability.

All reagents used in this study will be made available upon reasonable request to the corresponding author. The scRNA-seq data sets studied were previously published (39) and deposited in the NCBI under GEO dataset GSE254606. Values for all data points in the graphs are provided in the Supporting Data Values file.

## Author contributions

RK and BBG contributed to conceptualization, data curation, interpretation, formal analysis, funding acquisition, and writing the original draft. KN, BK, NC, TP, K Sharma, K Singh, CM, DFB, JN, AP, AM, LS, SK, and MHL performed data curation and formal analysis. ABM and KRS performed data interpretation and curation. DS and RMT contributed to data interpretation, analysis, and manuscript editing. MDG and TT performed data curation, analysis, and echocardiography in human participants. QP provided resources and supervised the entire human study and experiments related to human biospecimens.

## Supplementary Material

Supplemental data

Supporting data values

## Version 1 01/30/2025

In-Press Preview

## Version 2 03/17/2025

Electronic publication
