# Supplementary material for: Monocytes and interstitial macrophages contribute to hypoxic pulmonary hypertension
Source: J Clin Invest. 2025 Jan 30;135(6):e176865. doi: 10.1172/JCI176865 (PMC11910231; doi:10.1172/JCI176865)
Supplement: Supplemental data [file jci-135-176865-s050.pdf]

Supplementary Information

Monocytes and interstitial macrophages contribute to hypoxic pulmonary hypertension

Kumar R et. al.

Supplemental Table 1. Primers used for mouse mRNA quantification by RT-PCR.

All primers are TAQMAN Gene Expression Assays (Life Technologies Corporation, Carlsbad, CA, USA).

Target	Catalog #	Interrogated Sequence	Amplicon Length
Ccr2	Mm00438270_m1	NM_009915.2	100
Cx3cr1	Mm02620111_s1	NM_009987.4	107
Ccl2	Mm00441242_m1	NM_011333.3	74
Thbs1	Mm01335413_g1	NM_011580.3	84
Actb	Mm02619580_g1	AK075973.1	143

Supplemental Table 2: Antibodies used in the flow cytometry experiment of mouse biospecimen.

Antibody Specificity	Fluorochrome	Clone	Final Concentration	Manufacturer
anti-mouse CD16/32 (blocking Fc domain)	-	93	1µg	BioLegend
anti-mouse CD3	eFluo450	17A2	0.5µg	eBioscience®
anti-mouse CD19	eFluo450	1D3	0.5µg	eBioscience®
anti-mouse NK1.1	eFluo450	PK136	0.5µg	eBioscience®
anti-mouse Ly6G	eFluo450	1A8	0.5µg	eBioscience®
Fixable Viability dye	eFluo450		0.0625µg	eBioscience®
anti-mouse CD117 (c-Kit)	PE	2B8	0.125µg	BioLegend
anti-mouse CD135 (Flt3)	PE/Cyanine5	A2F10	0.25µg	eBioscience®
anti-mouse CD115 (CSF1R)	PE/eFluor 610	AFS98	0.5µg	eBioscience®
anti-mouse CD45	AF700	30 F11	1µg	eBioscience®
	BV570	30 F11	1µg	BioLegend
	BV785	30 F11	0.3µg	BioLegend
anti-mouse CD11b	SB600	M1/70	0.3µg	eBioscience®
	FITC	M1/70	0.125µg	BioLegend
anti-mouse CD64	PerCP/eFluor 710	X54-5/7.1	1µg	eBioscience®
	APC	X54-5/7.1	0.8µg	BioLegend
	PE/Cyanine7	X54-5/7.1	0.5µg	BioLegend
anti-mouse CD11c	PE/Cyanine7	N418	0.5µg	BioLegend
	APC	N418	0.3µg	BioLegend
anti-mouse Ly6C	APC	HK1.4	1µg	eBioscience®
	BV650	HK1.4	0.2µg	BioLegend
anti-mouse I-A/I-E	SB700	M5/114.15.2	0.15µg	eBioscience®
anti-mouse FOLR2	APC	10/FR2	0.6µg	BioLegend

anti-mouse CCR2	BV510	SA203G11	1µg	BioLegend
anti-mouse Ki-67	PE/Cyanine7	SolA15	0.4µg	eBioscience®
	AF700	16A8	0.5µg	BioLegend
anti-mouse TSP-1	PE	TX17.10	0.4µg	Novus
anti-mouse CCL2	FITC	2H5	0.3µg	eBioscience®
anti-mouse CD31	APC	390	0.5µg	BioLegend
anti-mouse Podoplanin (gp-38)	PE/Cyanine7	8.1.1	0.5µg	BioLegend

Supplemental Table 3: Surface marker signatures used in the flow cytometry experiment to characterize monocytes/ macrophages and their precursors in mouse BM, blood and lungs in the mouse samples.

Population	Compartment	Surface marker signature
Lin- (Dump)	BM, Blood and Lungs	(CD3 CD19 NK1.1 Ly6G VD)$^{-}$
Macrophage dendritic cells progenitors (MDP)	BM and Blood	Lin^{-} CD117^{+} CD115^{+} CD135^{+} Ly6C^{-} CD11b^{-}
Common monocytes progenitors (cMoP)	BM and Blood	Lin^{-} CD117^{+} CD115^{+} CD135^{-} Ly6C^{+} CD11b^{-}
Classical Monocytes (cMo)	BM and Blood	Lin^{-} CD117^{-} CD115^{+} CD135^{-} CD11b^{+} Ly6c^{hi}
Nonclassical Monocytes (ncMo)	BM and Blood	Lin^{-} CD117^{-} CD115^{+} CD135^{-} CD11b^{+} Ly6c^{low}
Alveolar Macrophages (AMs)	Lungs	Lin^{-} IVCD45^{-} CD64^{+} CD11b^{-}, CD11c^{+}, FOLR2^{-}
Classical monocytes derived Recruited IMs (cMo-IMs)	Lungs	Lin^{-} IVCD45^{-} CD64^{+} CD11b^{+} CD11c$^{int\text{-}hi}$ FOLR2^{-} CCR2^{+} CX3CR1^{-}
Nonclassical monocytes derived Recruited IMs (ncMo-IMs)	Lungs	Lin^{-} IVCD45^{-} CD64^{+} CD11b^{+} CD11c$^{int\text{-}hi}$ FOLR2^{-} CCR2$^{+/-}$ CX3CR1^{+}
FOLR^{+} Resident IMs	Lungs	Lin^{-} IVCD45^{-} CD64^{+} CD11b^{+} CD11c$^{int\text{-}hi}$ FOLR2^{+} CCR2$^{+/-}$ CX3CR1^{+}
Endothelial cells (ECs)	Lungs	Lin^{-} CD45^{-} CD31^{+} gp-38^{-}
Fibroblasts	Lungs	Lin^{-} CD45^{-} CD31^{-} gp-38^{+}
Other stromal cells	Lungs	Lin^{-} CD45^{-} CD31^{-} gp-38^{-}

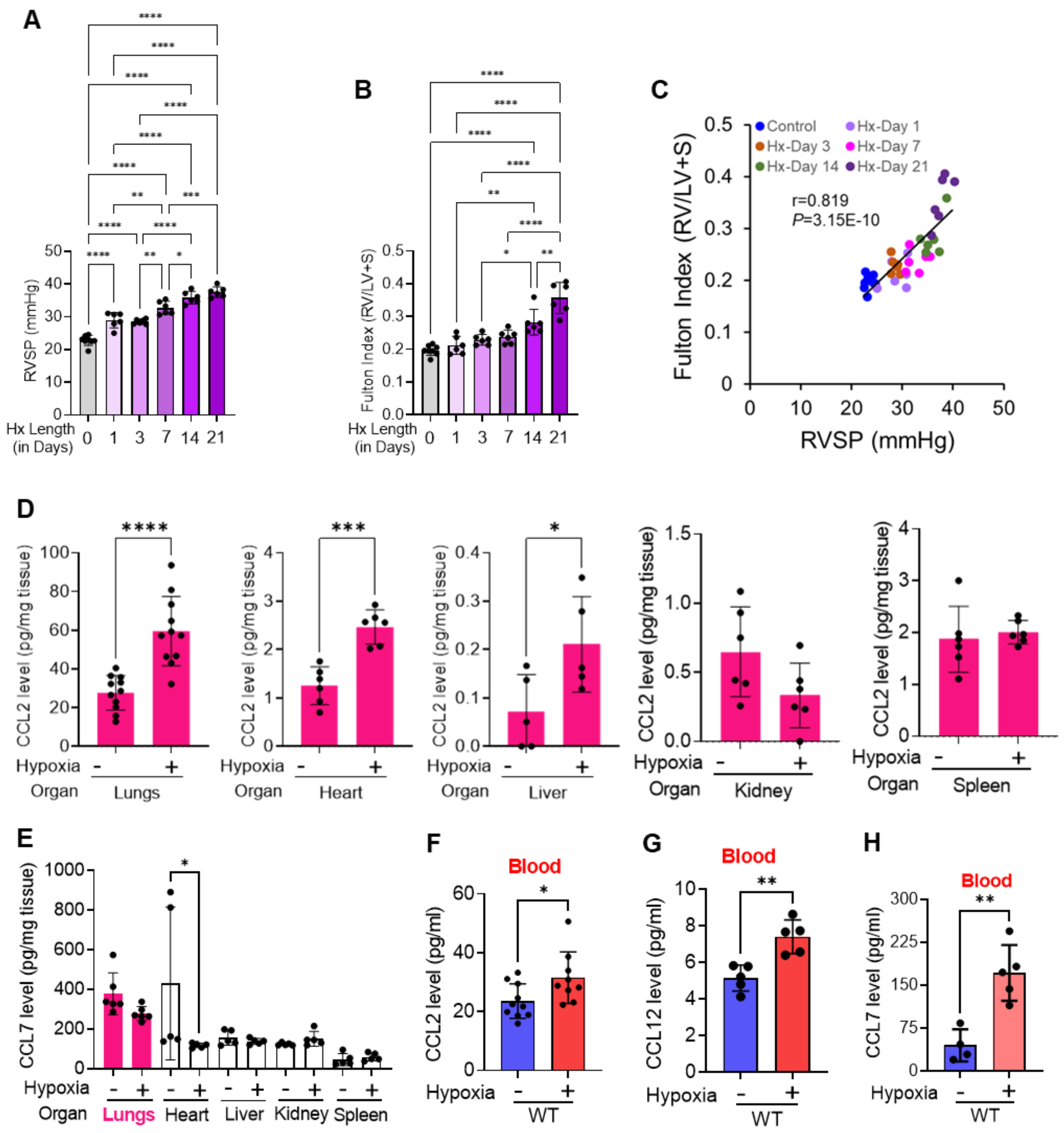

Supplemental Figure 1: Hypoxia exposure on days 1, 3, 7, 14, and 21 resulted in higher (**A**) right ventricular (RV) systolic pressure (RVSP) and (**B**) RV hypertrophy compared to normoxic controls (Day 0). A time course-based analysis showed a positive correlation between (**C**) RVSP and RV hypertrophy (Fulton index) following hypoxia exposure. (**D**) In an organ-specific independent comparison, the lungs, heart, and liver of wildtype mice exposed to hypoxia displayed elevated levels of CCL2 compared to the corresponding organs in normoxic wildtype mice. There was no difference in CCL7 levels in (**E**) whole lungs. Higher levels of (**F**) CCL2 (N=10/group), (**G**) CCL12 (N=5/group), and (**H**) CCL7 were observed in the intravascular compartment of wildtype mice exposed to hypoxia for 3 days compared to normoxic controls. A one-way ANOVA followed by Tukey's post hoc test was conducted for Panels A–B and E–H. Unpaired two-tailed t-tests were performed for Panels D–H. Data for Panels A–H were obtained from female wildtype mice. Statistical significance was indicated as follows: *P<0.05; **P<0.01; ***P<0.001; ****P<0.0001.

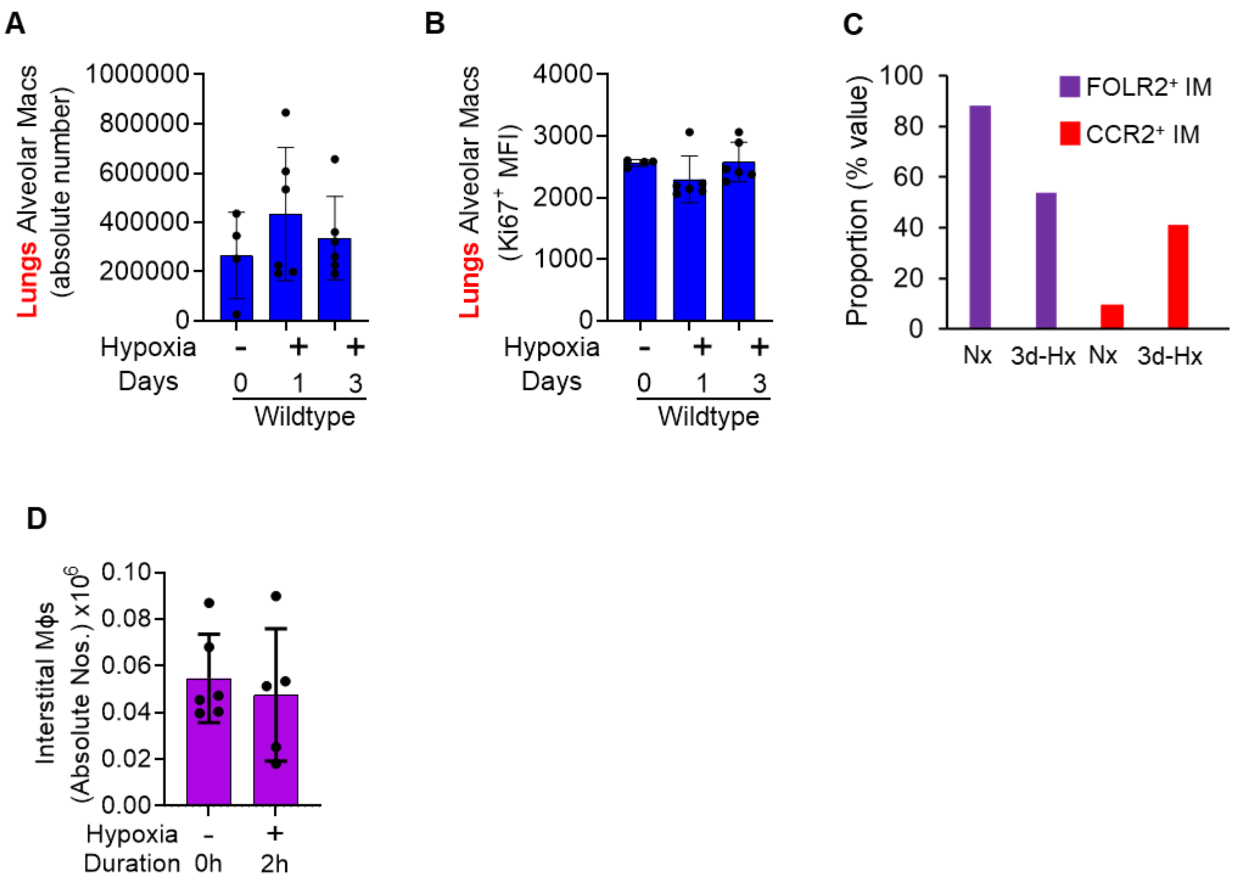

Supplemental Figure 2: Flow cytometry-based timepoint analysis showed no significant difference in the (**A**) absolute number or (**B**) proliferation of alveolar macrophages between unexposed and hypoxia-exposed wildtype mice. (**C**) A reduced proportion of FOLR2+ IMs in the lungs was observed due to an increased influx of CCR2+ IMs after 3 days (3d) of hypoxia exposure. A short duration of 2-hour hypoxia exposure (**D**) showed no difference in interstitial macrophages between normoxia and 2-hour hypoxia in wildtype mice, as assessed by flow cytometry. IMs: interstitial macrophages. Nx: normoxia; Hx: hypoxia. Data were obtained from female mice and were normally distributed. ANOVA followed by Tukey's post hoc test was used to compare more than two groups, while t-tests were performed for comparisons between two groups. Results are presented as mean ± SD. MFI: median fluorescence intensity.

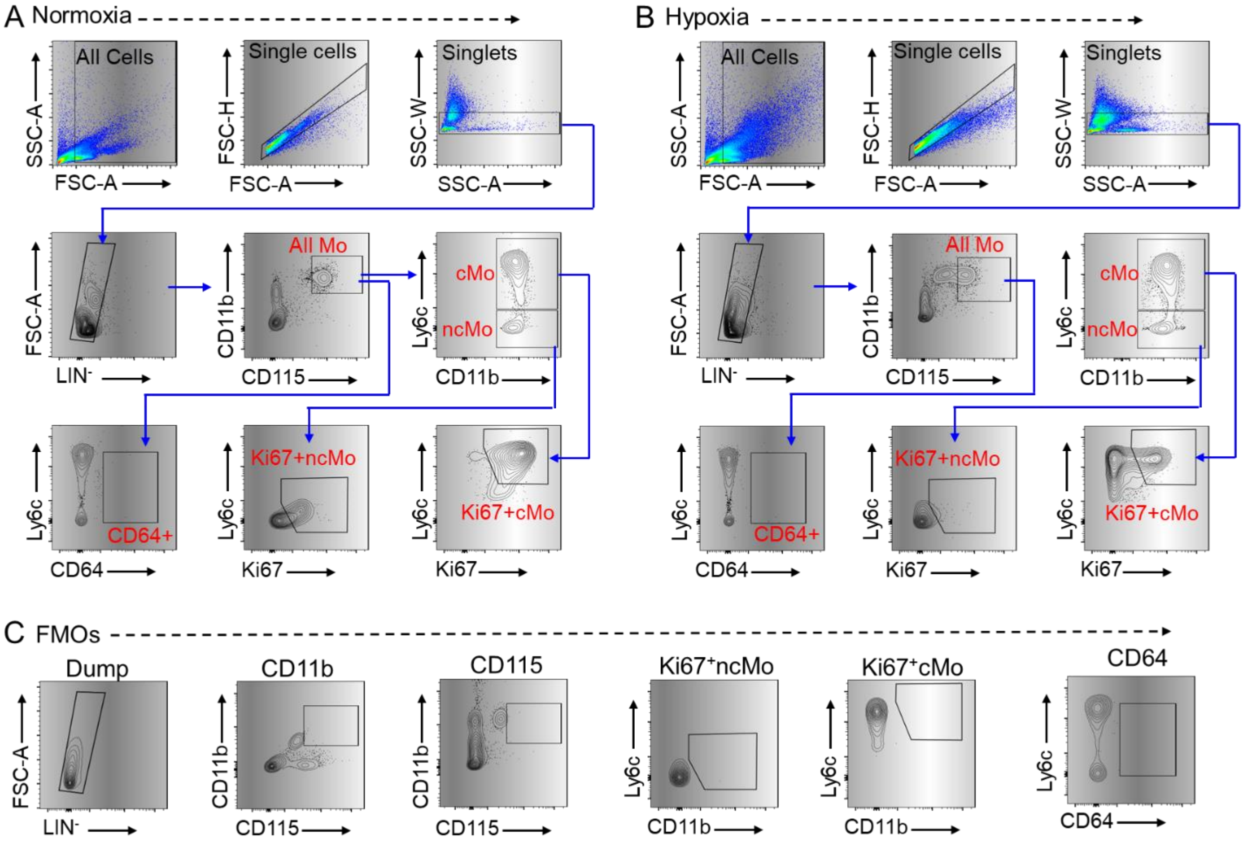

Supplemental Figure 3: Flow cytometry gating strategy for monocyte characterization in the intravascular compartment. Monocytes (CD11b+CD115+) and their subtypes were identified as follows: classical monocytes (cMo), characterized as CD11b+CD115+Ly6Chi, and nonclassical monocytes (ncMo), characterized as CD11b+CD115+Ly6C− within the Lin− (CD3−CD19−NK1.1−Ly6G−VD−) population. This analysis was performed using PBMCs from (**A**) normoxia- and (**B**) hypoxia-exposed wildtype mice. (**C**) Fluorescence-minus-one (FMO) controls were used to establish gating thresholds for these cells. Additional parameters included SSC-A (side scatter area), FSC-A (forward scatter area), and SSC-W (side scatter width). VD represents the viability dye used to exclude dead cells.

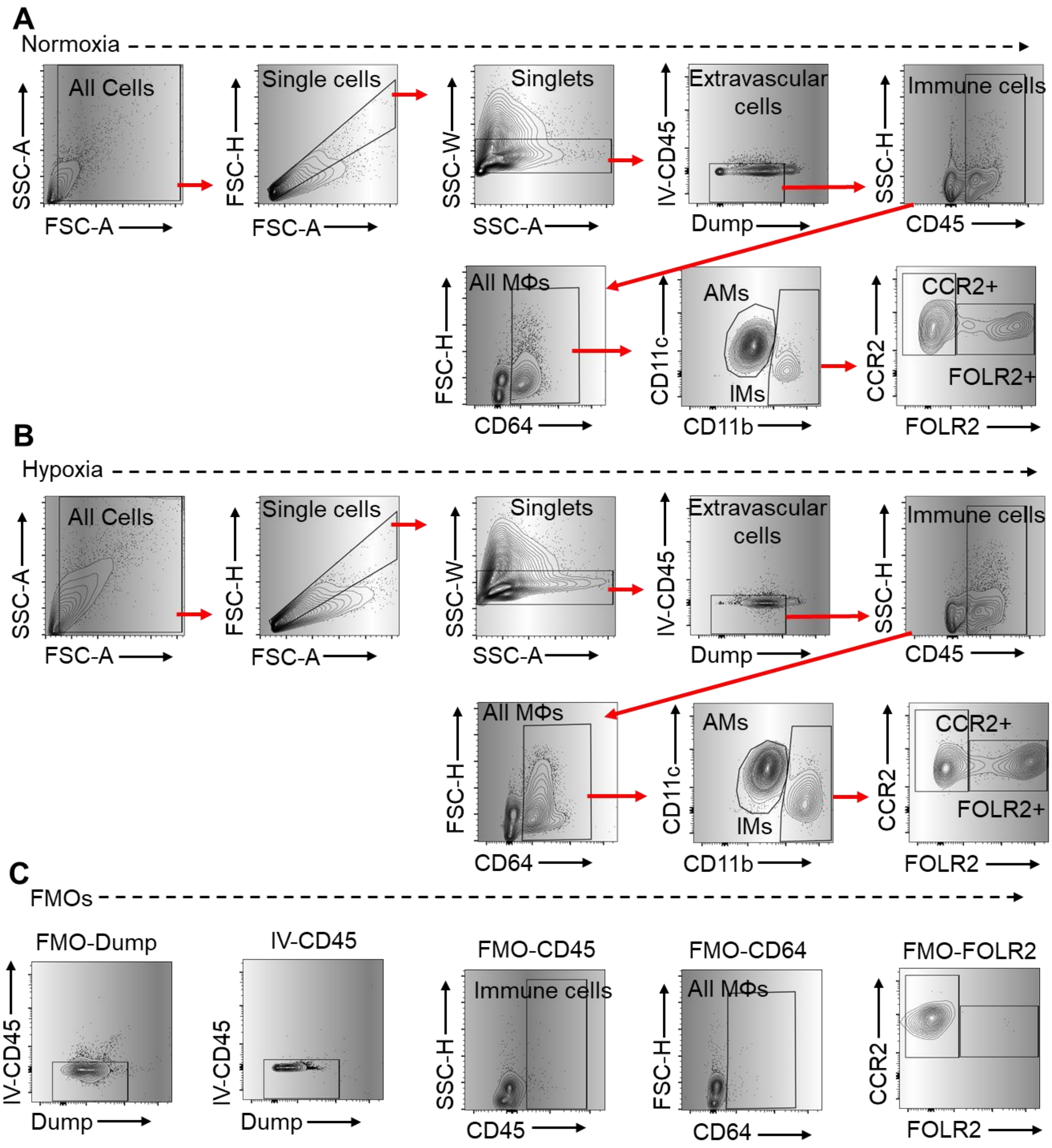

Supplemental Figure 4. Schematic representation of lung macrophages populations of normoxia and hypoxia-exposed wildtype mice. (**A**) Gating strategy for flow cytometry of normoxic (**B**) Hypoxia (vehicle) with (**C**) FMOs. Briefly, singlets were gated after removing the doublets from all the cells based on forward scatter -area versus -height and side scatter -area versus -width parameters. Singlets were further gated to removed dump (T cells, B cells, NK cells, neutrophils, and dead cells) and intravascular cells (negative for intravenous CD45) to select extravascular immune cells (CD45+). Extravascular cells were further gated to separate all macrophages (CD64+ subsets). These macrophages were further differentiated into alveolar macrophages (AMs) and interstitial macrophages (IMs). IMs were further characterized into recruited (CCR2+) and resident (FOLR2+) populations.

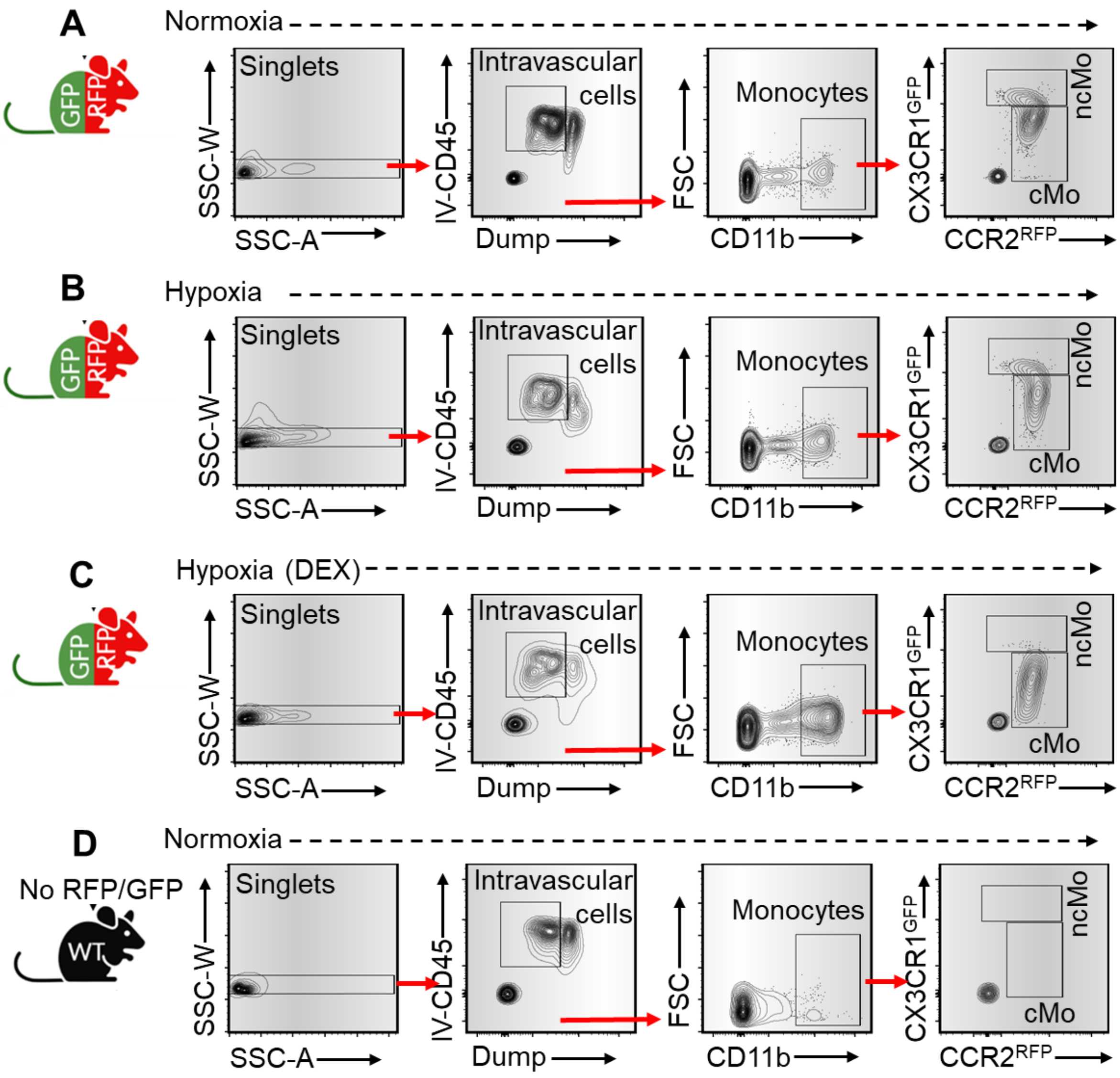

Supplemental Figure 5: Gating strategy using PBMCs to track intravascular monocytes in the normoxic and hypoxia-exposed double reporter mice. (**A-C**) Gating strategy to characterize the CCR2+ classical monocytes and CX3CR1+ nonclassical monocytes in normoxia (control) and hypoxia exposed PBS and DEX treated double reporter with GFP and RFP expression: singlets were gated for dump- and IVCD45+ to identify the intravascular immune cells. Intravascular cells were further gated to separate all monocytes (CD11b+ subsets). These macrophages were further characterized into CCR2+ classical based on RFP expression and nonclassical CX3CR1+ based on higher GFP expression. (**D**) wildtype mice PBMCs (no RFP or GFP signal) represent a control for double reporter mice.

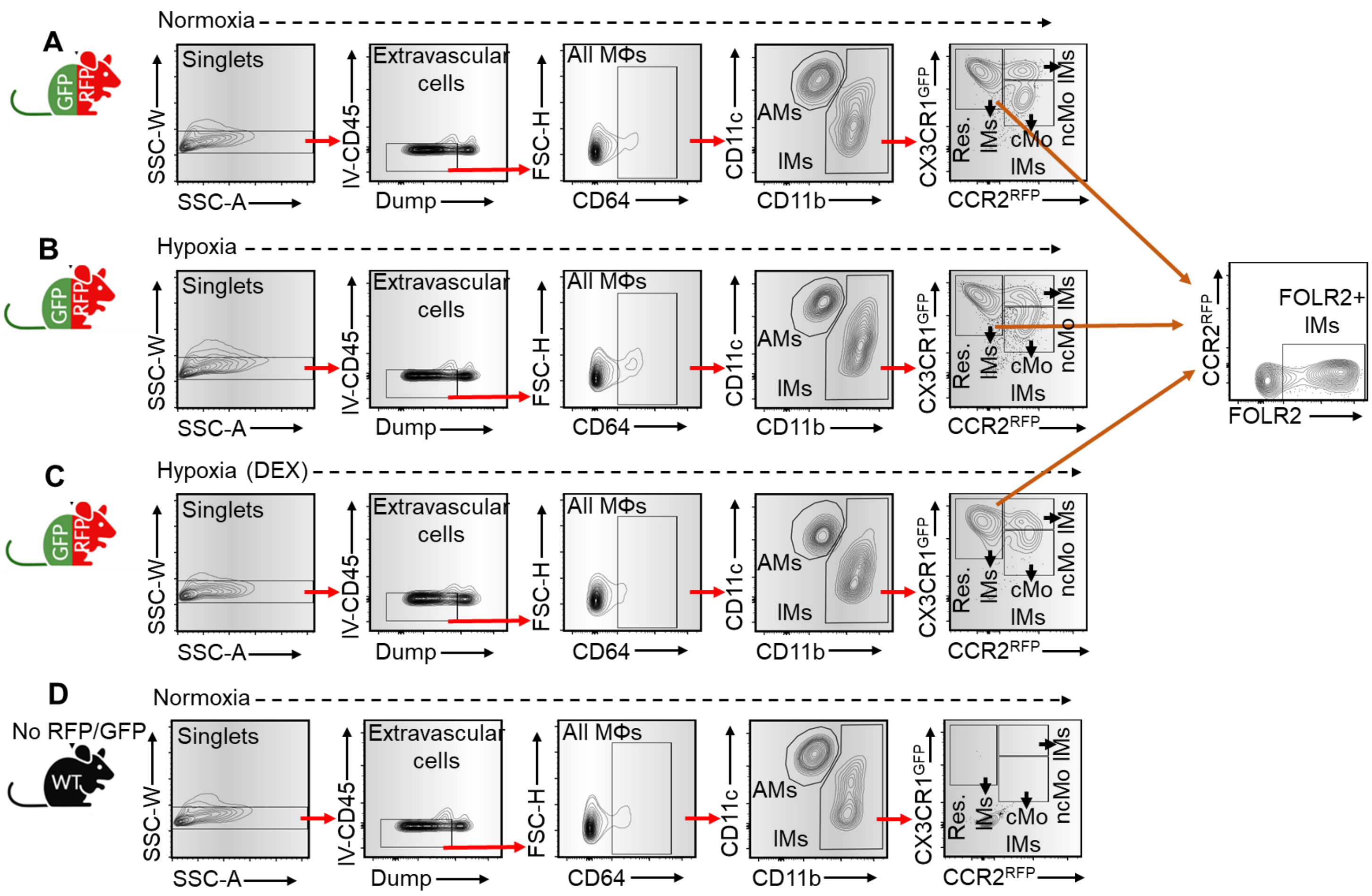

Supplemental Figure 6: Schematic representation of flow cytometry analysis showing TSP-1+ cells using unexposed and hypoxia-exposed double reporter and wildtype mice. (**A-C**) Gating strategy to characterize the recruited and resident IMs in normoxia (control) and hypoxia exposed PBS and DEX treated double reporter with GFP and RFP expression: singlets were gated for RFP+ cells, followed by identifying the extravascular immune cells. Extravascular cells were further gated to separate all macrophages (CD64+ subsets). These macrophages were further differentiated into AMs and IMs based on CD11b and CD11c surface markers. IMs were further characterized into recruited (CCR2+) and resident populations (FOLR2). (**D**) wildtype mice (no RFP or GFP signal) represent a control for double reporter mice.

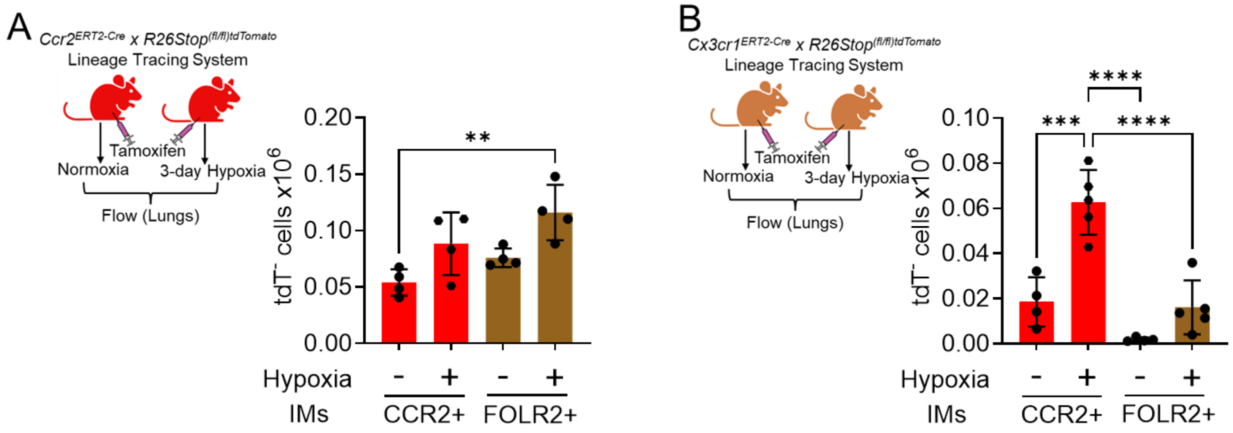

Supplemental Figure 7. Tdt- gates from *Cx3cr1*$^{ERT2\text{-}Cre}$ *x R26Stop*$^{(fl/fl)tdTomato}$ and *Ccr2*$^{ERT2\text{-}Cre}$ *x R26Stop*$^{(fl/fl)tdTomato}$ lineage tracing systems to study the origins of recruited and resident IMs. 3 days of hypoxia exposure following one week following tamoxifen administration using (**A**) *Cx3cr1*$^{ERT2\text{-}Cre}$ *x R26Stop*$^{(fl/fl)tdTomato}$ showed a significant increase in tdT$^-$ cells within the CCR2+ IMs (N=4-5/ group; 2M, 2F in Normoxia; 2M and 3F in Hypoxia group). Whereas, with similar condition, (**B**) *Ccr2*$^{ERT2\text{-}Cre}$ *x R26Stop*$^{(fl/fl)tdTomato}$ showed that increase in FOLR2+ IMs was largely from tdT$^-$ populations, while there was little change in tdT$^-$ CCR2+ IMs with hypoxia exposure (N=4/ group; 2M, 2F in normoxia; 2M and 3F, 1M in Hypoxia group). ANOVA followed by Tukey's post hoc test was conducted. mean ± SD plotted. **P<0.01, ***P<0.001, ****P<0.0001.

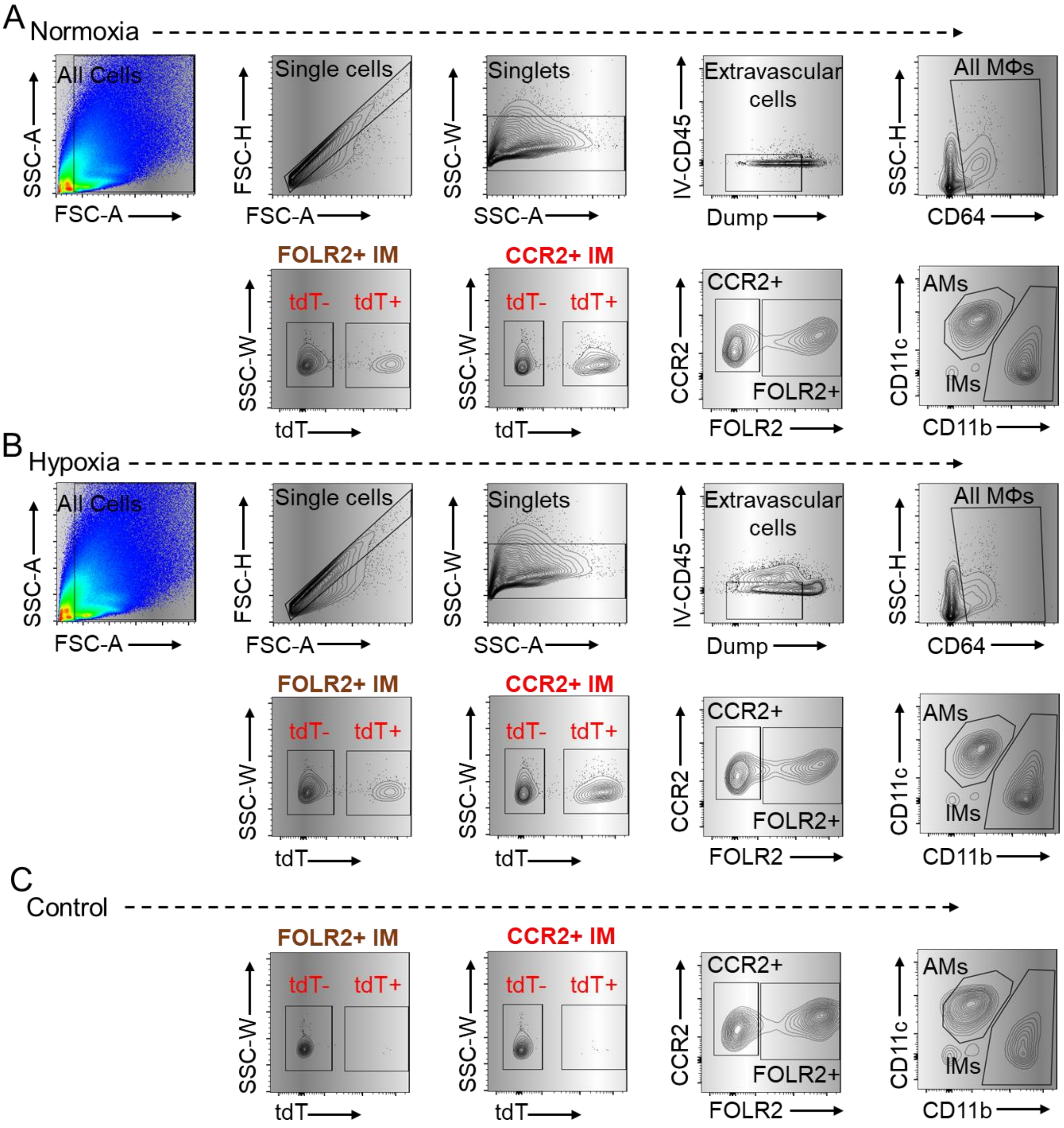

Supplemental Figure 8. *Ccr2*$^{ERT2\text{-}Cre}$ *x R26Stop*$^{(fl/fl)tdTomato}$ pulse-chase lineage tracing system. Tamoxifen administration efficiently labels resident CCR2+ IMs with tdT without marking resident FOLR2+ IMs. Briefly, both in (**A**) normoxia and (**B**) hypoxia, singlets were gated for dump and intravascular CD45 negative subsets to identify extravascular immune cells. Extravascular cells were then gated to isolate macrophages (CD64+ subsets), which were further categorized into alveolar macrophages (AMs) and IMs based on CD11b and CD11c surface markers. IMs were subsequently divided into recruited (CCR2+) and resident (FOLR2+) populations, with tdT expression confirming their origin. (**C**) wildtype mice were used as a negative control.

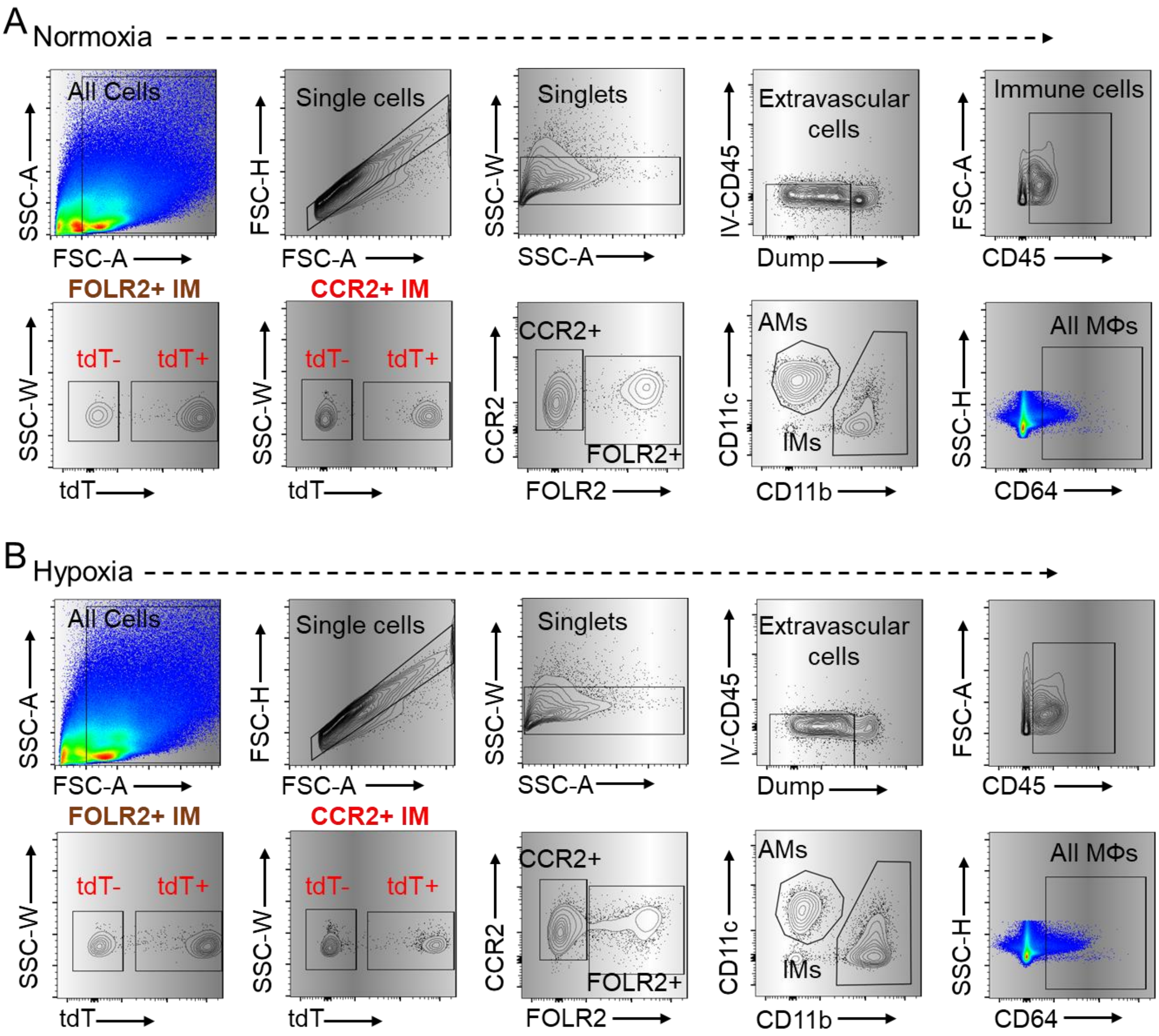

Supplemental Figure 9. *Cx3cr1*$^{ERT2\text{-}Cre}$ *x R26Stop*$^{(fl/fl)tdTomato}$ pulse-chase lineage tracing system. Tamoxifen administration efficiently labels resident FOLR2+ IMs with tdTomato (tdT) without marking circulating CCR2+ monocytes. Briefly, both in (**A**) normoxia and (**B**) hypoxia, singlets were gated for the dump and intravascular CD45 negative subsets to identify extravascular immune cells. Extravascular cells were then gated to isolate CD45+ immune cells followed by identifying all macrophages (CD64+ subsets). These CD64+ cells were further categorized into alveolar macrophages (AMs) and IMs based on CD11b and CD11c surface markers. IMs were subsequently divided into recruited (CCR2+) and resident (FOLR2+) populations, with tdT expression confirming their origin.

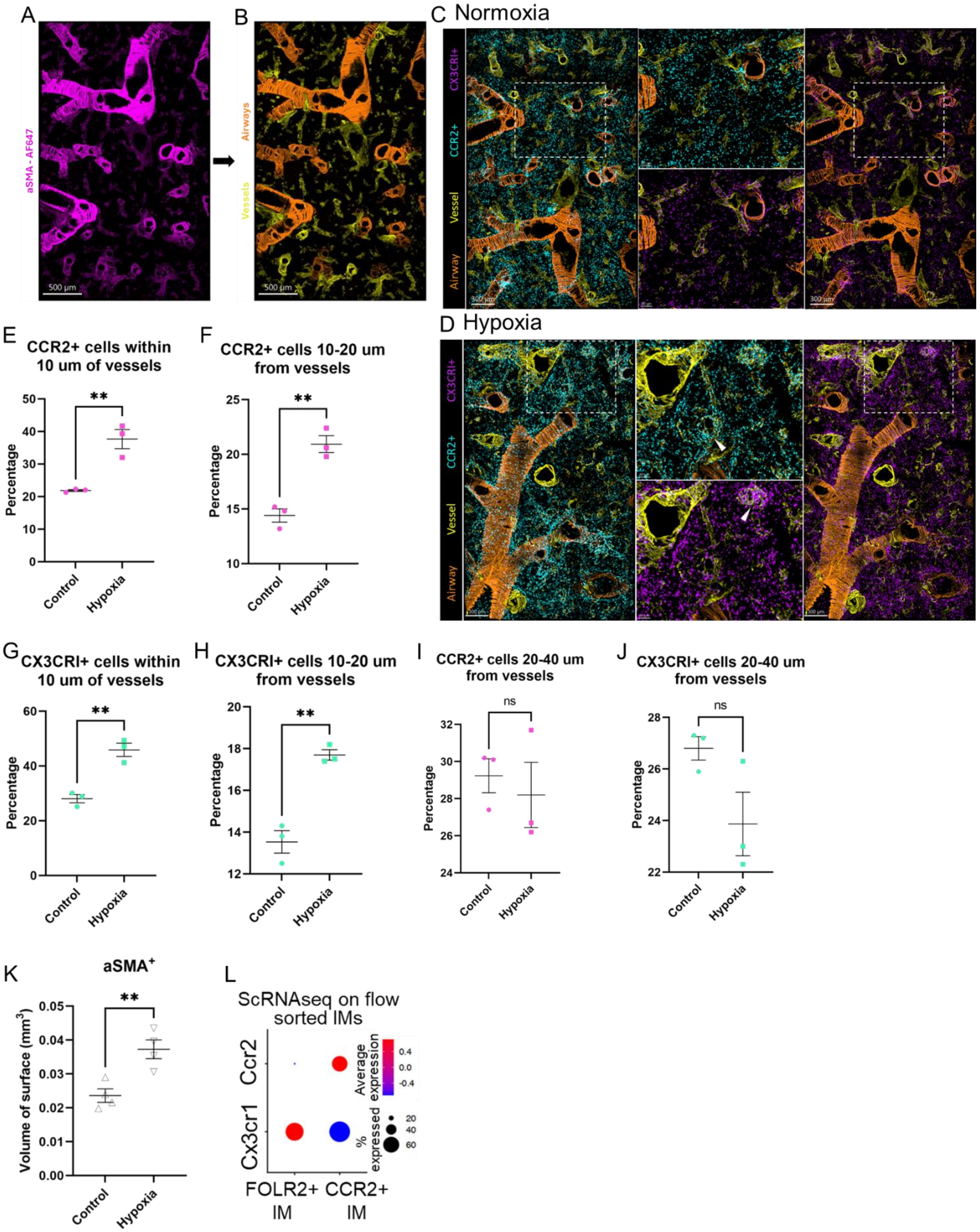
A
B
C Normoxia
D Hypoxia
aSMA - AF647
500 µm
Airways
Vessels
CX3CRI+
CCR2+
Vessel
Airway
300 µm
E
CCR2+ cells within 10 um of vessels
F
CCR2+ cells 10-20 um from vessels
G
CX3CRI+ cells within 10 um of vessels
H
CX3CRI+ cells 10-20 um from vessels
I
CCR2+ cells 20-40 um from vessels
J
CX3CRI+ cells 20-40 um from vessels
Percentage
Control
Hypoxia
**
ns
K
aSMA+
Volume of surface (mm3)
L
ScRNAseq on flow sorted IMs
Ccr2
Cx3cr1
FOLR2+ IM
CCR2+ IM
Average expression
% expressed

Supplemental Figure 10. Qualitative and quantitative imaging of Cx3cr1gfp-Ccr2rfp reporter mice reveals increased density of IM subsets in the perivascular region after hypoxia exposure. Vessels and airways were analyzed by creating a surface of the (**A**) αSMA channel, and then (**B**) airways and vessels were manually segmented based on morphology. Recruited IMs expressing $CCR2^+$ and non-recruited $FOLR2^+$ resident IMs expressing CX3CR1 in (**C**) normoxic (control) and (**D**) 3-day hypoxia exposed lungs were quantified. A significant increase in the percentages of (**E & F**) $CCR2^+$ and (**G & H**) $CX3CR1^+$ cells within 10 μm and 10–20 μm distances from the vessels in hypoxic lungs, compared to normoxic lungs, was detected. (**I & J**) There was no difference in both $CCR2^+$ and $CX3CR1^+$ cells within 20-40um regions. (**K**) There was media remodeling as shown by significantly increased aSMA volume in the hypoxic lungs compared to the control lungs. (**L**) Recently published single-cell RNA sequencing (scRNA-seq) data from sorted IMs of normoxia- and 3-days hypoxia-exposed mice demonstrated higher *Cx3cr1* expression in resident FOLR2+ IMs, while *Ccr2* was predominantly expressed by recruited CCR2+ IMs[39]. Scale bars of 500μm for the low magnification images. Data were collected from female mice, and statistical analysis was performed using unpaired two-tailed t-tests. mean ± SD plotted; $^{**}P < 0.01$.

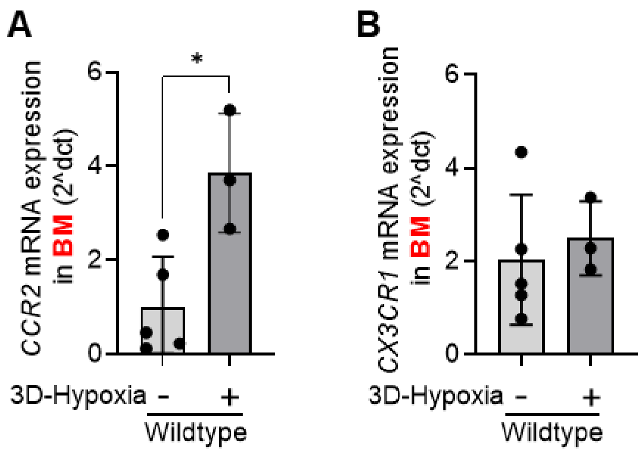

Supplemental Figure 11. *Ccr2* and *Cx3cr1* mRNA expression in the BM compartment. qPCR analysis showed a significantly higher expression for (**A**) the classical monocyte receptor, Ccr2, whereas there was no difference in (**B**) the nonclassical monocyte receptor, Cx3cr1 in the 3 days hypoxia exposed wildtype mice compared to unexposed mice. $2^{-\Delta Ct}$ relative to β-*actin* housekeeping gene; n=3/5 per group; Data were normally distributed. mean ± SD plotted; Unpaired two-tailed t-tests were performed. *P<0.05, ****P<0.001. BM, bone marrow.

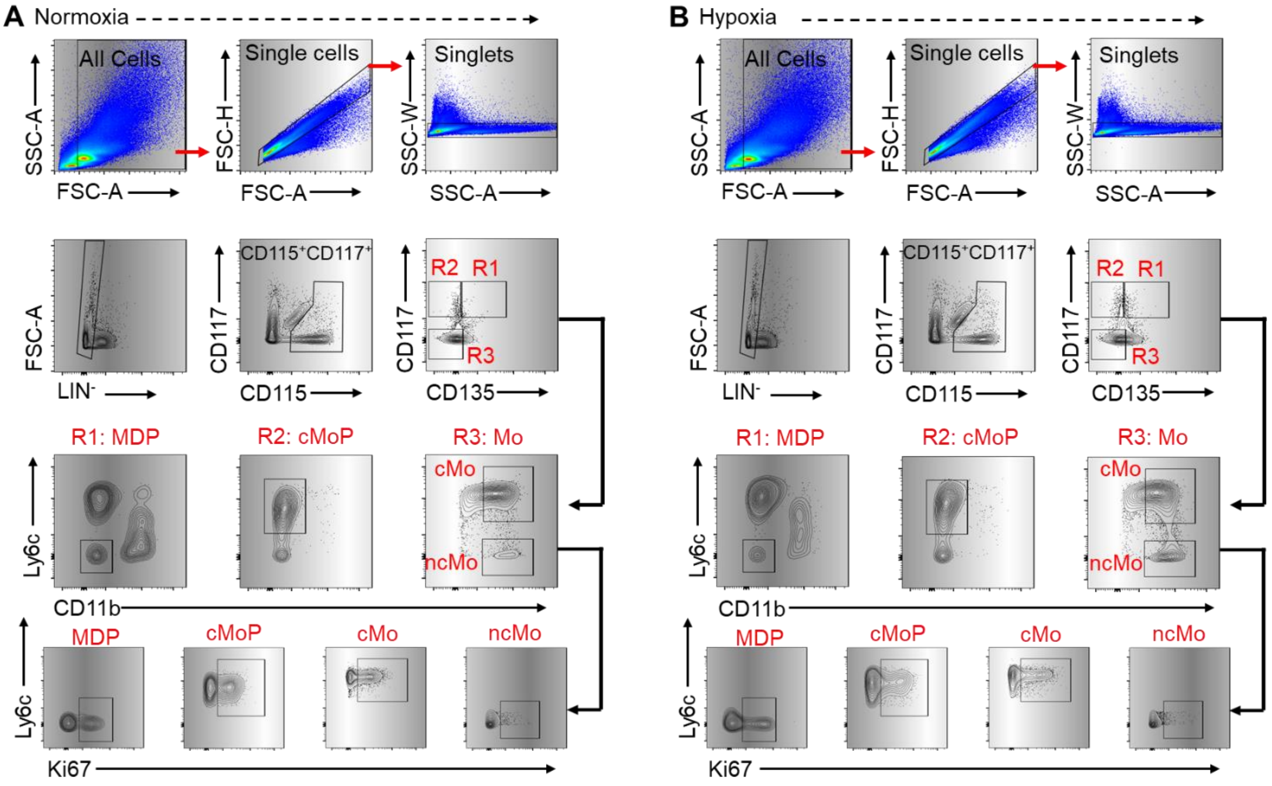

Supplemental Figure 12. Flow cytometry gating of monocytes and its precursors' cells in BM. MDP (R1: CD117+CD115+CD135+Ly6C−CD11b−), cMoP cells (R2: CD117+CD115+CD135−Ly6C+CD11b−), and monocytes (Mo; R3: CD117−CD115+CD135−Ly6Chi or Ly6CloCD11b+) were identified within the Lin− (CD3−CD19−NK1.1−Ly6G−VD−) bone marrow compartment of (**A**) normoxia- and (**B**) hypoxia-exposed wildtype mice. Additional parameters included SSC-A (side scatter area), FSC-A (forward scatter area), and SSC-W (side scatter width). VD represents the viability dye used to exclude dead cells.

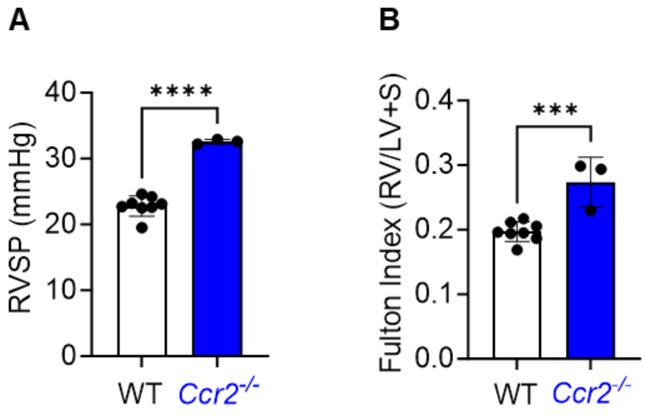

Supplemental Figure 13. Global *Ccr2*$^{-/-}$ mice have at baseline higher PH phenotype. Unexposed *Ccr2*$^{-/-}$ mice showed higher (**A**) right ventricular systolic pressure (RVSP) and (**B**) Fulton index (RV /(LV+S)) compared to wildtype mice (N=3-8/group). Data were obtained from female mice and were normally distributed. Unpaired two-tailed t-tests were performed. mean ± SD plotted. ***P<0.001, ****P<0.0001. WT, wildtype; RV, Right ventricle; LV, left ventricle; S, septum.

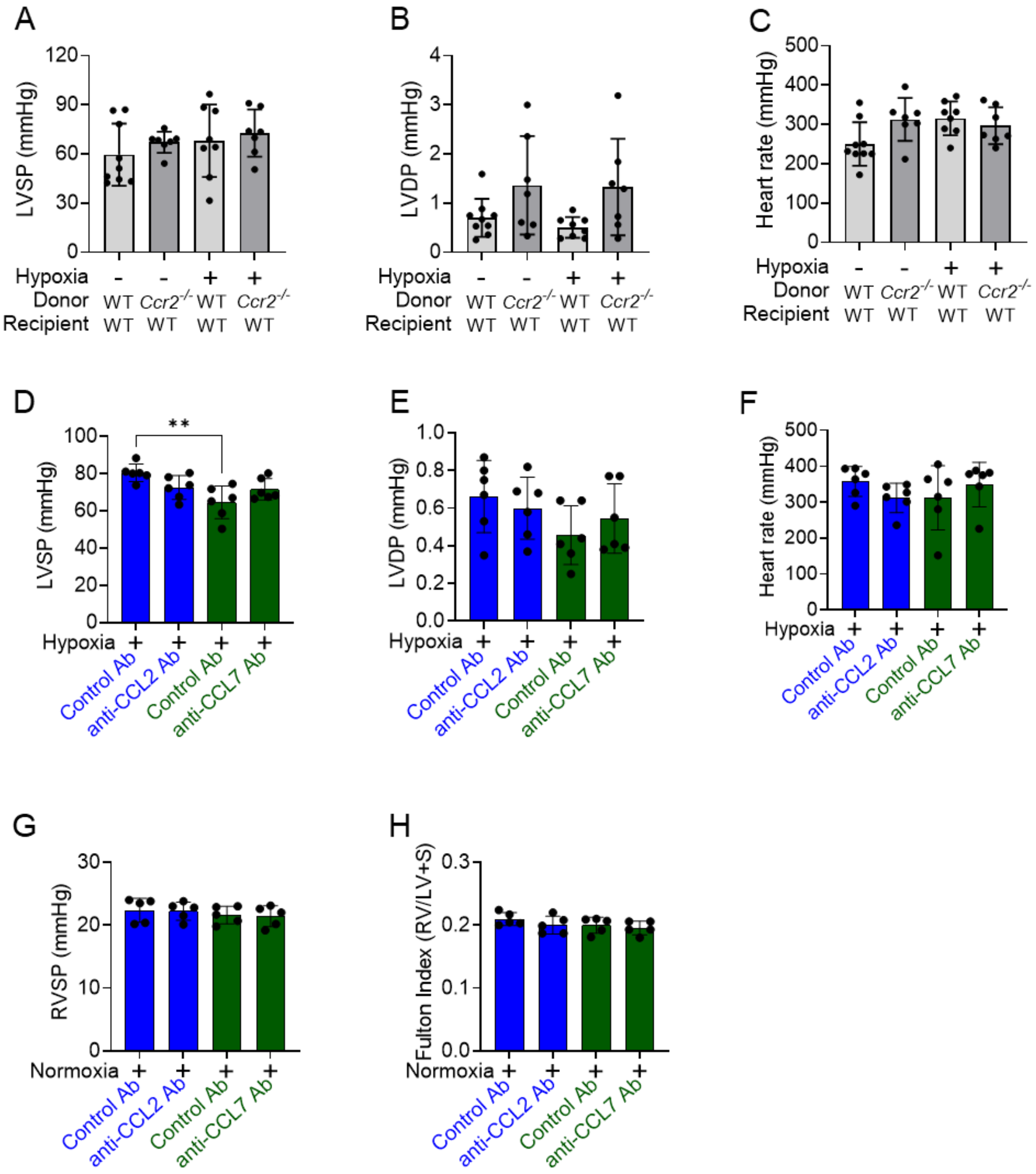

Supplemental Figure 14. Blockade of classical monocytes receptor or ligands showed no difference in the left heart hemodynamics parameters. (A-C) Hypoxia exposed wildtype mice receiving either *Ccr2*$^{-/-}$ BM or **(D-F)** wildtype mice receiving either CCL2 or CCL7 neutralizing antibody (NAb) shows no difference in Left ventricular systolic pressure (LVSP), left ventricular diastolic pressures (LVDP) and heart rate compared to hypoxia-exposed wildtype mice receiving wildtype BM (N=7-8/group) or wildtype mice receiving control antibodies (N=6/group), respectively. (**G and H**) No effect of anti-CCL2 or anti-CCL5 neutralizing antibody on the baseline phenotypes of normoxic wildtype mice. Data were obtained from female mice. ANOVA followed by Tukey's post hoc test was used to compare more than two groups. ***P*<0.01. WT, wildtype; Ab, antibody; LVSP, left ventricular systolic pressure; LVDP, left ventricular diastolic pressure; HR, heart rate, RVSP, right ventricle systolic pressure; RV, right ventricle; LV, left ventricle, S, septum.

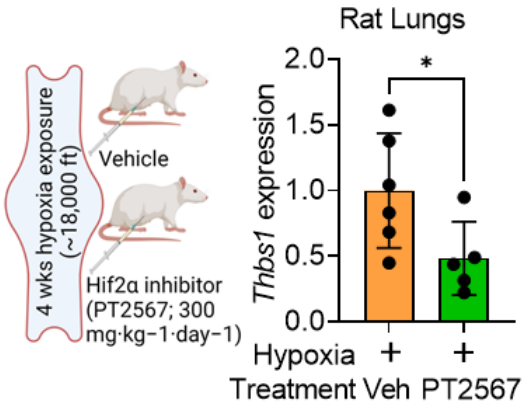

Supplemental Figure 15. Blockade of Hif2α using small molecule inhibitor significantly attenuates *Thbs1* expression in the lungs of hypoxic Rats (N=5-6/group). Data were generated from female rats, and were normally distributed. Unpaired two-tailed t-tests were performed. mean ± SD plotted. *$P<0.05$.

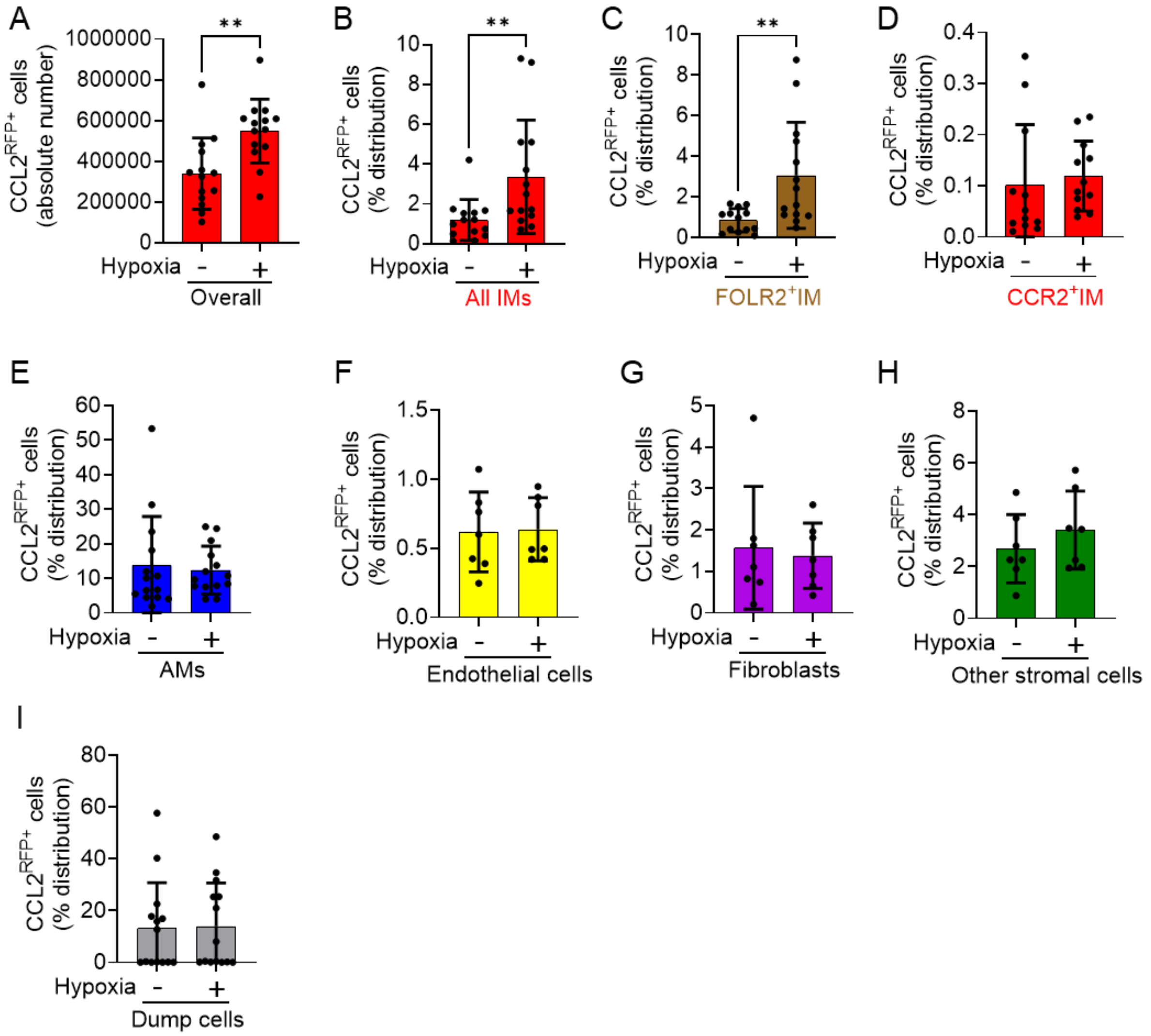

Supplemental Figure 16. Distribution of cells in normoxia and hypoxia exposed CCL2-RFP mice, for (**A**) all CCL2^{RFP+} cells (**B**) all IMs, (**C**) resident FOLR2^{+} IMs, (**D**) recruited CCR2^{+}IMs, (**E**) alveolar macrophages (AMs), (**F**) endothelial cells, (**G**) fibroblasts, (**H**) other stromal cells (not ECs or fibroblasts), and (**I**) dump gate cells (including B cells, T cells, NK cells, neutrophils and dead cells). n=7-14/group. Data were not normally distributed; therefore, for all panels, Mann-Whitney U tests were performed. mean ± SD plotted. *P<0.05, **P<0.01.

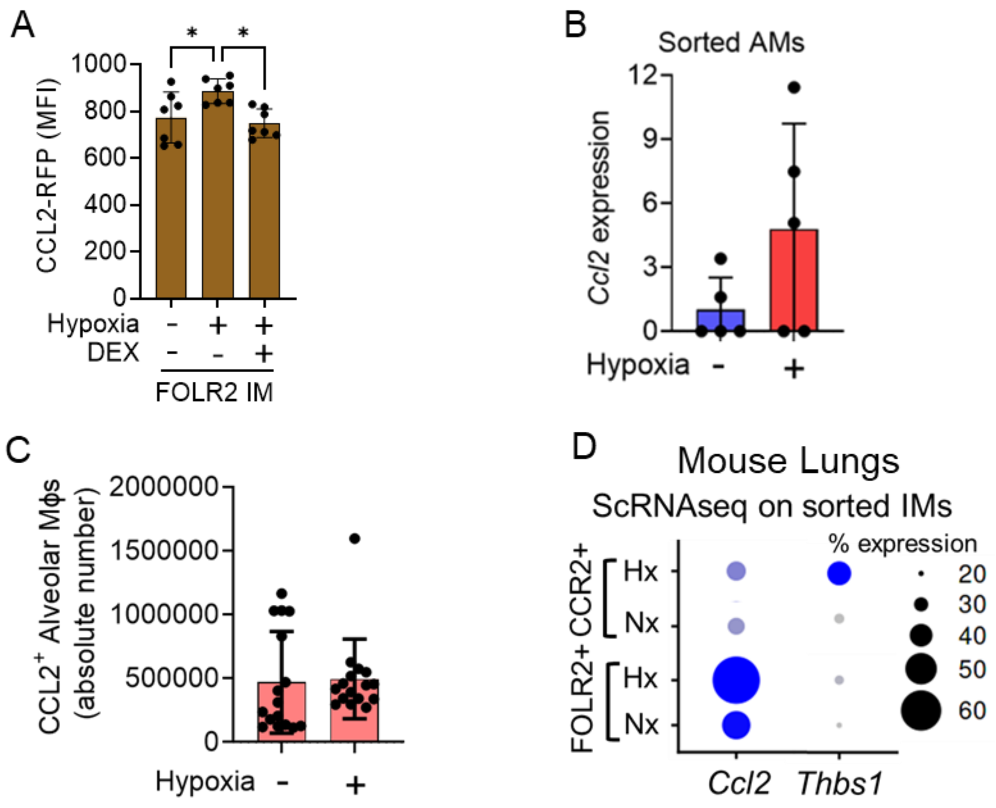

Supplemental Figure 17. (**A**) Resident FOLR2+ IMs exhibited higher CCL2 MFI following hypoxia exposure; however, treatment with DEX attenuated CCL2 expression, as demonstrated using *Ccl2*$^{RFP\text{-}floxl}$ reporter mice. (**B**) No significant change in *Ccl2* expression in bronchoalveolar lavage-sorted alveolar macrophage (AM) populations. (**C**) No change in the number of CCL2RFP+ AMs following hypoxia exposure. (**D**) Published single-cell RNA sequencing (scRNA-seq) data from sorted interstitial macrophages (IMs) of normoxia- and hypoxia-exposed mice demonstrated higher *Ccl2* expression in resident FOLR2+ IMs, while *Thbs1* was predominantly expressed by recruited CCR2+ IMs[39]. Data in panels A and B were not normally distributed; therefore, a Mann-Whitney U test was performed to compare the two groups. Data are presented as mean ± SD, *P<0.05. MFI, mean fluorescence intensity; DEX, dexamethasone; AM, alveolar macrophages.

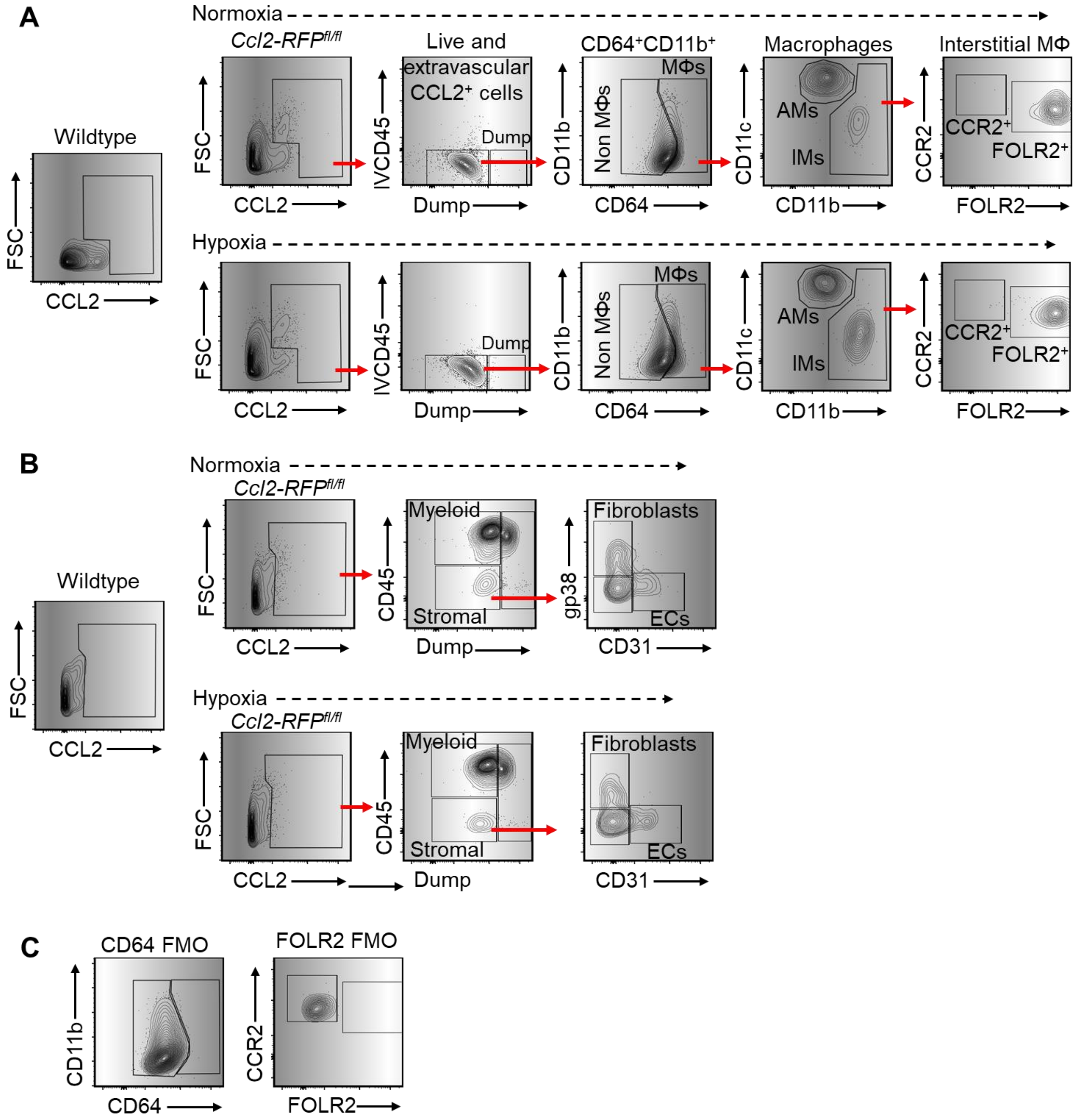

Supplemental Figure 18. Schematic representation of flow cytometry analysis showing CCL2+ cells using unexposed and hypoxia exposed CCL2^{RFP} reporter mice. (**A**) Gating strategy for flow cytometry: singlets were gated for RFP+ cells, followed by identifying the extravascular immune cells. Intravascular cells were further gated to separate all macrophages (CD64+ subsets). These macrophages were further differentiated into AMs and IMs. IMs were further characterized into recruited and resident populations. Similarly (**B**), RFP+ stromal cells were characterized using endothelial cells (ECs) and stromal cells pan markers CD31 and gp-38, respectively (threshold for RFP+ cells were determined by wildtype mice (non RFP+ cells). (**C**) FMOs control for CD64 and FOLR IMs; representative image of 7-14 / group).

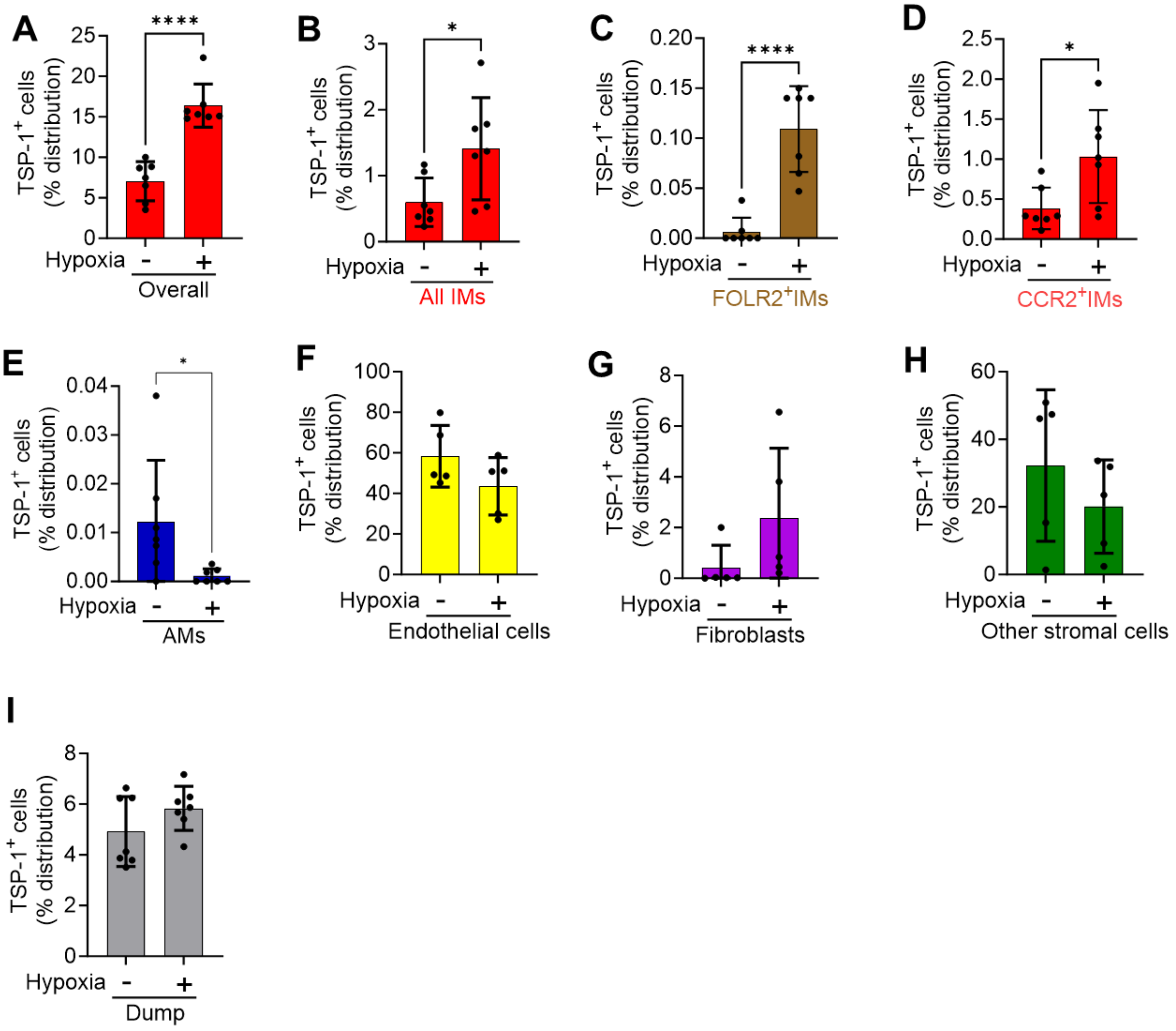

Supplemental Figure 19. Distribution of TSP-1+ cells in normoxia and 3 days hypoxia exposed wildtype mice. The distribution of TSP-1$^+$ lung cells in normoxia and hypoxia exposed wildtype mice of (**A**) all TSP-1$^+$ cells, (**B**) all IMs, (**C**) resident FOLR2$^+$ IMs (only 2 normoxic samples identified any TSP-1$^+$ FOLR2$^+$ IMs), (**D**) recruited CCR2$^+$IMs, (**E**) alveolar macrophages (AMs), (**F**) endothelial cells, (**G**) fibroblasts, (**H**) other stromal cells (not ECs or fibroblasts) and (**I**) dump gate cells (B cells, T cells, NK cells, neutrophils and dead cells); n=7/group. Unpaired two-tailed t-tests were performed. mean ± SD plotted. *P<0.05, ****P<0.0001.

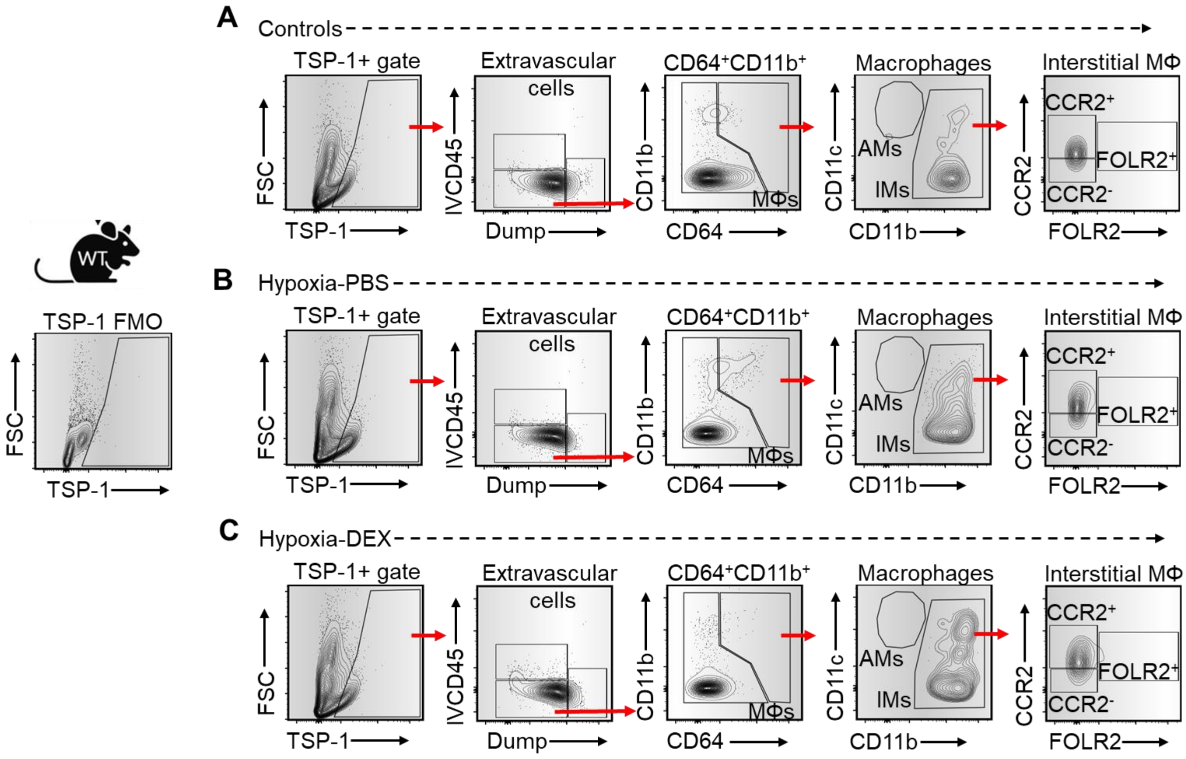

Supplemental Figure 20. Schematic representation of flow cytometry analysis showing TSP-1+ cells using unexposed and hypoxia-exposed wildtype mice. (**A-C**) Gating strategy to characterize immune cells in normoxia (control) and hypoxia exposed PBS and DEX treated mice: singlets were gated for TSP-1+ cells, followed by identifying the extravascular immune cells. Extravascular cells were further gated to separate all macrophages (CD64+ subsets). These macrophages were further differentiated into AMs and IMs. IMs were further characterized into recruited and resident populations.

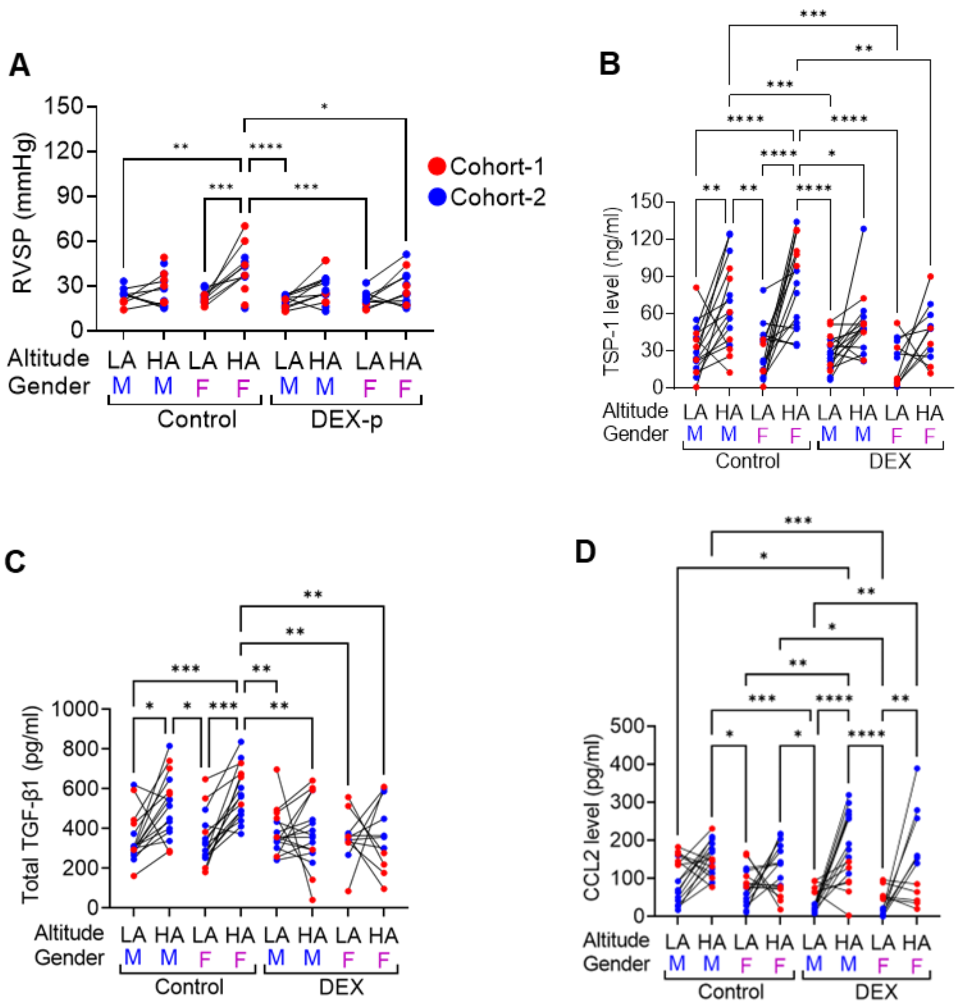

Supplemental Figure 21: Sex based analysis on the effect of DEX prophylaxis on RVSP and inflammatory proteins in the blood. Effect of 3 days HA exposure without DEX (control) and with DEX prophylaxis (DEX-p) on (**A**) RVSP and inflammatory proteins (**B**) TSP-1 and (**C**) TGF-β1 and (**D**) CCL2 levels. The data were normally distributed. Statistical analysis was performed using ANOVA, followed by Tukey's post hoc test. mean ± SD plotted. $^{\#}$*P*<0.05, ***P*<0.01, ****P*<0.001, *****P*<0.0001.

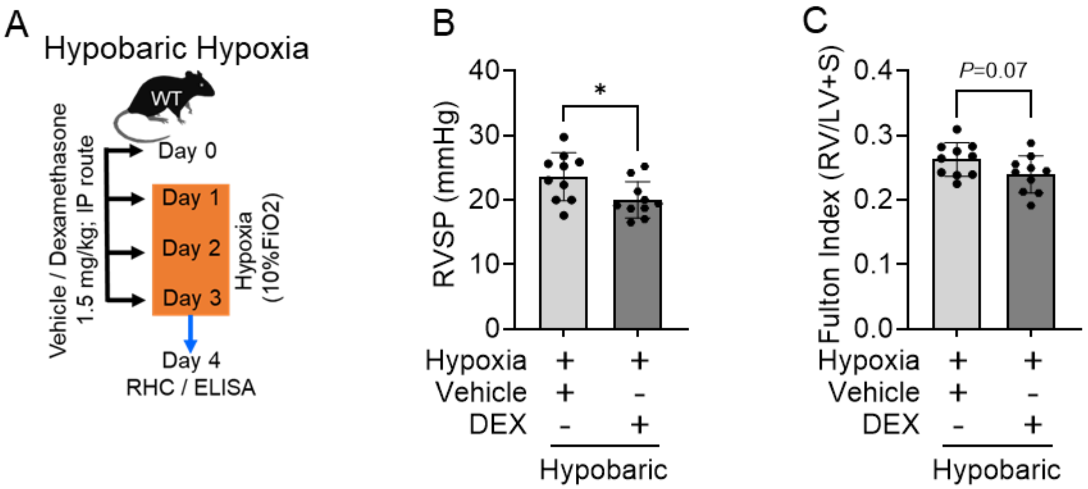

Supplemental Figure 22: DEX prophylaxis protects from hypobaric hypoxic PH. (**A**) Schematic showing the experimental design and the dosing of DEX prophylaxis. Hypobaric hypoxia-exposed wildtype mice treated with DEX prophylaxis had lower (**B**) RVSP and (**C**) a trend towards blunted RV hypertrophy (N=10/group). Data were generated from female mice, and were normally distributed. Unpaired two-tailed t-tests were performed. mean ± SD plotted. **P*<0.05. RVSP, right ventricular systolic pressure; RV, right ventricle; DEX, Dexamethasone; RVSP, right ventricle systolic pressure; RV, right ventricle; LV, left ventricle, S, septum.

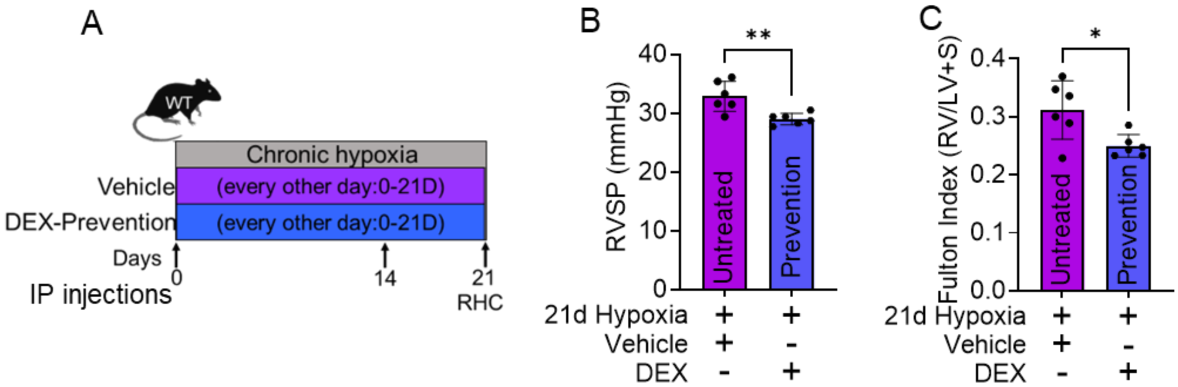

Supplemental Figure 23: DEX prophylaxis protects from chronic hypoxic PH. (**A**) Schematic showing the experimental design and the dosing of DEX prophylaxis. 21-d (day) hypoxia-exposed wildtype mice treated with DEX prophylaxis had lower (**B**) RVSP and (**C**) RV hypertrophy by RHC (N=6/group). Data were generated from female mice, and were normally distributed. Unpaired two-tailed t-tests were performed. mean ± SD plotted. $^{\#}P<0.05$, $^{**}P<0.01$. IP, intraperitoneal injection; RVSP, right ventricle systolic pressure; RV, right ventricle; LV, left ventricle, S, septum; DEX, Dexamethasone. RHC, right heart catheterization.

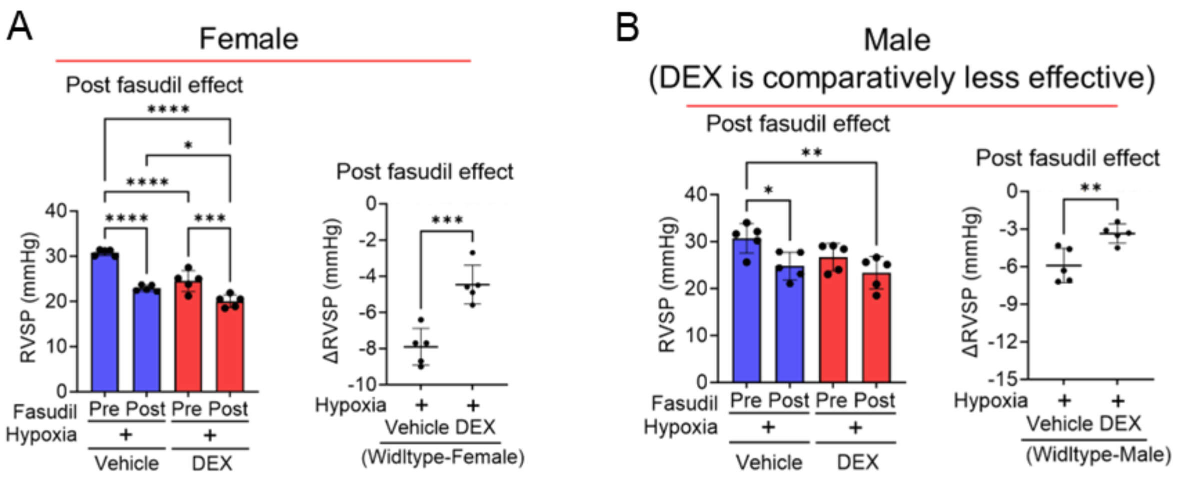

Supplemental Figure 24: DEX decreases Rho-kinase signaling and vasoconstriction induced by hypoxia with a greater effect in (**A**) females than (**B**) males. Data were normally distributed. ANOVA followed by Tukey's post hoc test was used to compare more than two groups, while t-tests were performed for comparisons between two groups. mean ± SD plotted. $^{\#}P$<0.05, **P<0.01, ***P<0.001, ****P<0.0001.

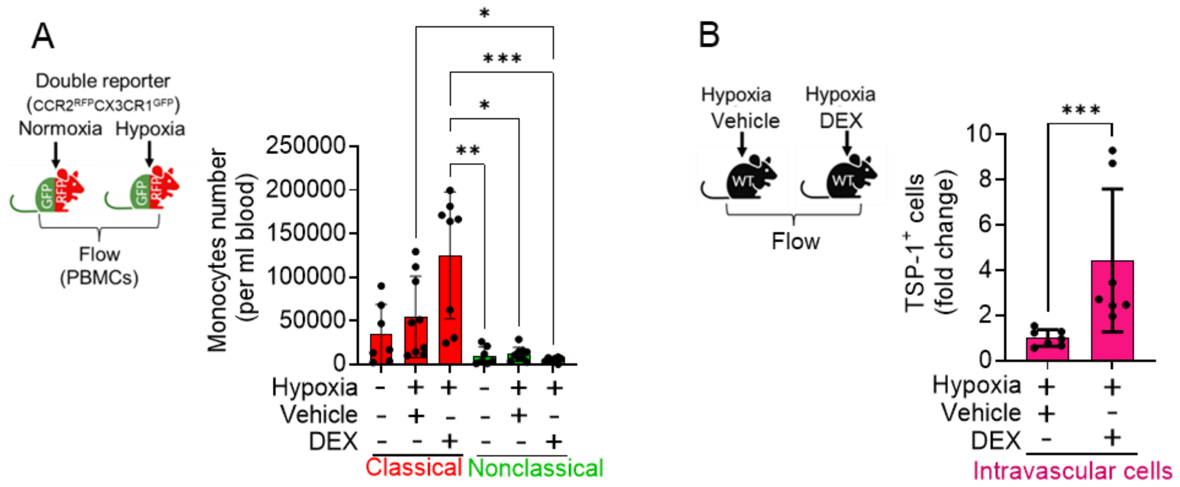

Supplemental Figure 25: Flow cytometry analysis exhibited a trend towards more intravascular monocytes in DEX-treated mice exposed to hypoxia for 3 days compared to hypoxia-exposed mice with vehicle treatment, as demonstrated in (**A**) *Ccr2*^{RFP}*Cx3cr1*^{GFP} double reporter mice. Moreover, these intravascular cells in wildtype mice showed elevated expression of (**B**) TSP-1, consistent with a failure to enter the lung tissue. Mean ± SD plotted. Data were not normally distributed; therefore, the Kruskal-Wallis ANOVA test, followed by Dunn's post hoc test, was used to compare more than two groups (Panel A). Mann-Whitney U tests (Panel B) were performed for comparisons between the two groups. $^{\#}P<0.05$, $^{**}P<0.01$, $^{***}P<0.001$, $^{****}P<0.0001$. DEX, dexamethasone; PBMCs: peripheral blood mononuclear cells.

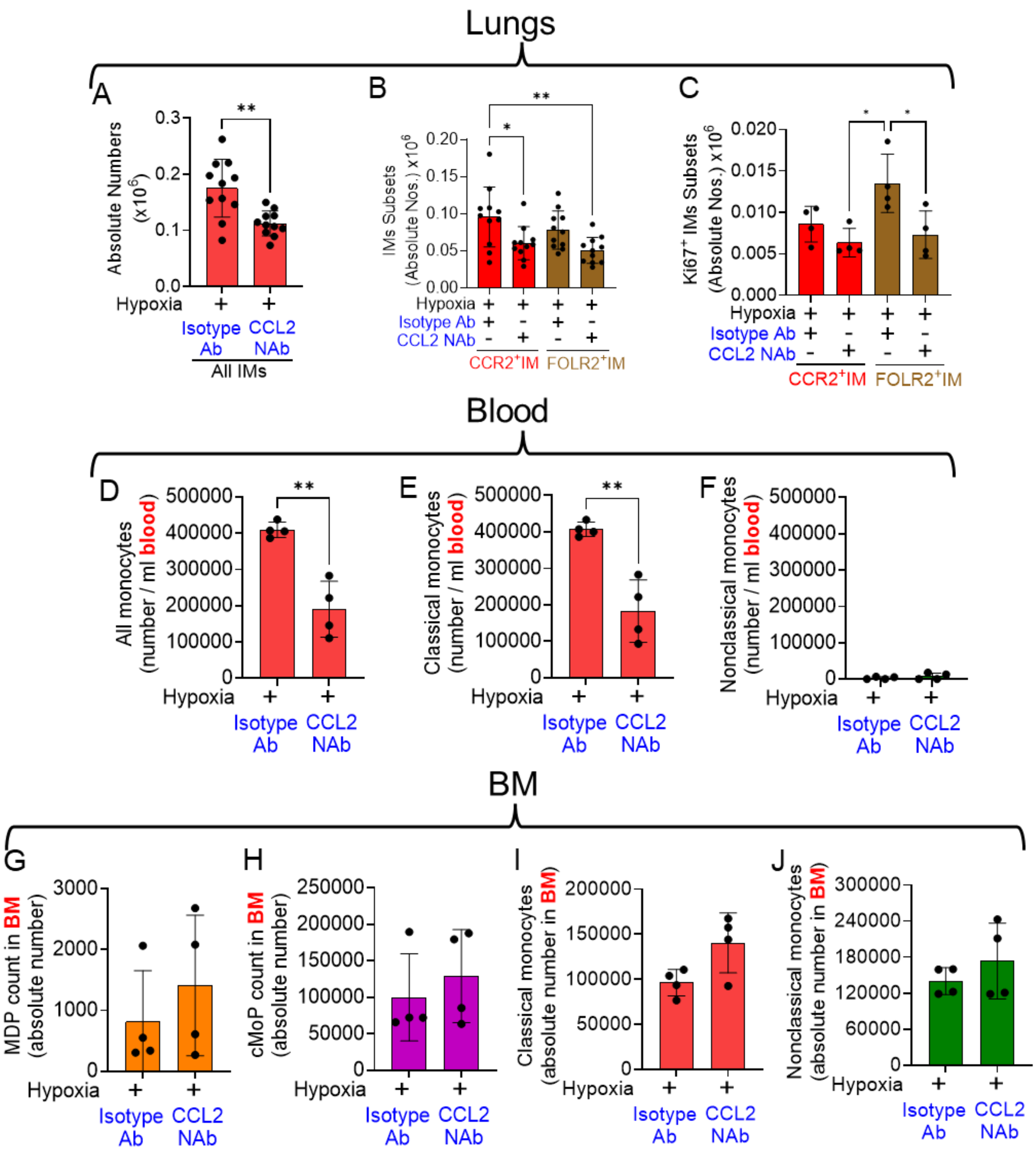

Supplemental Figure 26. Effect of anti-CCL2 NAb on lungs, blood, and BM compartment. Anti-CCL2 NAb-treated hypoxic mice showed a lower number of overall IMs, with attenuated recruitment of CCR2+ IMs and no recruitment of FOLR+ IMs, along with decreased proliferation (**A-C**) in the lungs. A reduced number of monocytes (**D-F**) was observed in the intravascular compartments. (**G-J**) A trend towards higher classical monocytes (cMo) in the BM was noted, with no changes in cMo progenitor cells in the BM of hypoxia-exposed wild-type mice treated with anti-CCL2 NAb. For panels A-E and I-J, data were normally distributed. ANOVA followed by Tukey's post hoc test was used for comparisons among more than two groups, while t-tests were performed for comparisons between two groups. Data in panels F-H were not normally distributed; therefore, Mann-Whitney U tests were performed for comparisons between the two groups. $^{\#}P<0.05$, $^{**}P<0.01$, $^{***}P<0.001$.

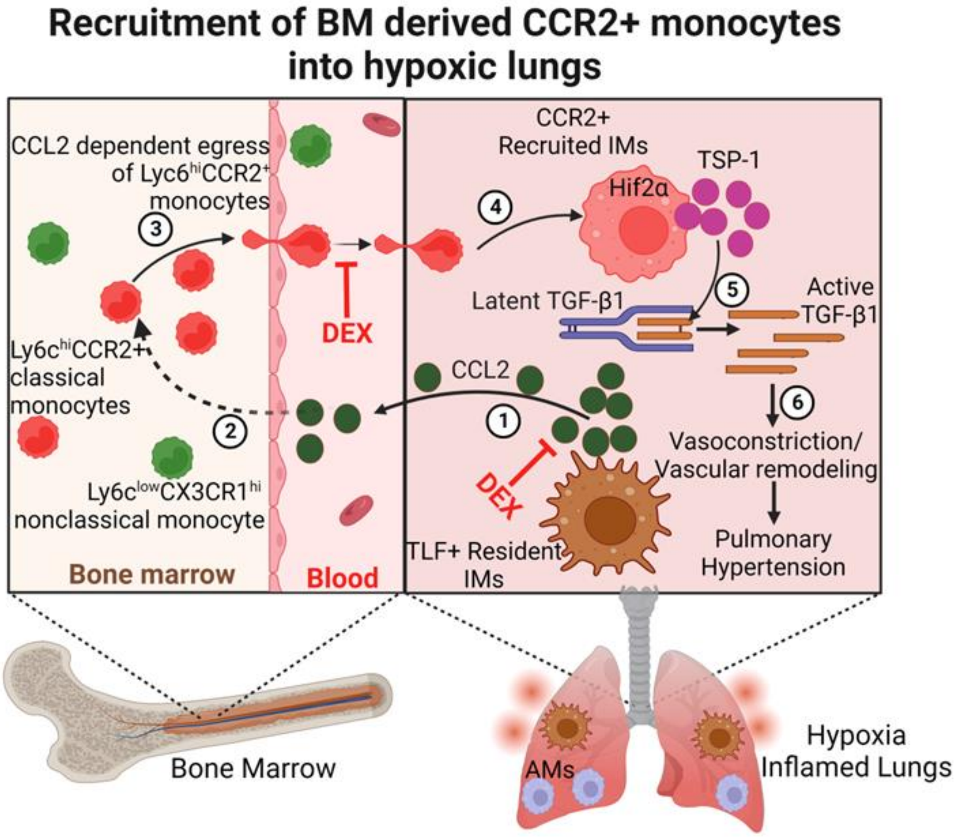

Supplemental Figure 27. Summary of signaling pathways. As outlined in the schematic, under hypoxic conditions, FOLR2^{+} resident IMs (depicted as TLF+) sense low oxygen levels and produce classical monocyte ligands, including CCL2 (step 1). These cytokines signal to the bone marrow through the circulation (step 2) to release CCR2+ monocytes into the bloodstream (step 3). Monocytes are then recruited into the lungs due to a higher concentration gradient of CCL2 emanating from the inflamed lungs, and upon entering the lungs they become interstitial macrophages (IMs) (step 4). Once recruited, these IMs release thrombospondin-1, a pathological protein that activates latent TGF-β1 (step 5). The active TGF-β1 causes rho kinase-mediated vasoconstriction and vascular remodeling (step 6), driving pulmonary hypertension. DEX prophylactic treatment abrogates the CCL2 gradient and prevents the recruitment of TSP-1 producing inflammatory IMs precursors CCR2+ monocytes into the lungs, ultimately protecting against hypoxic-PH.
